# Threshold-dependent repression of *SPL* gene expression by miR156/miR157 controls vegetative phase change in *Arabidopsis thaliana*

**DOI:** 10.1371/journal.pgen.1007337

**Published:** 2018-04-19

**Authors:** Jia He, Mingli Xu, Matthew R. Willmann, Kevin McCormick, Tieqiang Hu, Li Yang, Colby G. Starker, Daniel F. Voytas, Blake C. Meyers, R. Scott Poethig

**Affiliations:** 1 Department of Biology, University of Pennsylvania, Philadelphia, Pennsylvania, United States of America; 2 Department of Soil and Plant Sciences, University of Delaware, Newark, Delaware, United States of America; 3 Department of Genetics, Cell Biology and Development and Center for Genome Engineering, University of Minnesota, Minneapolis, Minnesota, United States of America; ANU, AUSTRALIA

## Abstract

Vegetative phase change is regulated by a decrease in the abundance of the miRNAs, miR156 and miR157, and the resulting increase in the expression of their targets, *SQUAMOSA PROMOTER BINDING PROTEIN-LIKE* (*SPL*) transcription factors. To determine how miR156/miR157 specify the quantitative and qualitative changes in leaf morphology that occur during vegetative phase change, we measured their abundance in successive leaves and characterized the phenotype of mutations in different *MIR156* and *MIR157* genes. miR156/miR157 decline rapidly between leaf 1&2 and leaf 3 and decrease more slowly after this point. The amount of miR156/miR157 in leaves 1&2 greatly exceeds the threshold required to specify their identity. Subsequent leaves have relatively low levels of miR156/miR157 and are sensitive to small changes in their abundance. In these later-formed leaves, the amount of miR156/miR157 is close to the threshold required to specify juvenile vs. adult identity; a relatively small decrease in the abundance of miR156/157 in these leaves produces a disproportionately large increase in SPL proteins and a significant change in leaf morphology. miR157 is more abundant than miR156 but has a smaller effect on shoot morphology and *SPL* gene expression than miR156. This may be attributable to the inefficiency with which miR157 is loaded onto AGO1, as well as to the presence of an extra nucleotide at the 5' end of miR157 that is mis-paired in the miR157:SPL13 duplex. miR156 represses different targets by different mechanisms: it regulates *SPL9* by a combination of transcript cleavage and translational repression and regulates *SPL13* primarily by translational repression. Our results offer a molecular explanation for the changes in leaf morphology that occur during shoot development in *Arabidopsis* and provide new insights into the mechanism by which miR156 and miR157 regulate gene expression.

## Author summary

Leaves produced at different stages in the development of an *Arabidopsis* shoot vary predictably in shape and size. Previous studies have shown that this phenomenon is regulated by variation in the abundance of the miRNAs, miR156 and miR157, but how miR156/miR157 produce the changes in leaf morphology that occur during shoot development is not understood. To answer this question, we measured the abundance of miR156/miR157 and their SPL targets in successive leaf primordia, and characterized the effect of variation in the abundance of miR156/miR157 on leaf morphology and the abundance of SPL transcripts and SPL proteins. miR156/miR157 are present at very high levels in the first two rosette leaves, where they act as buffers to stabilize leaf identity. They are present at lower and steadily declining levels in subsequent leaves, where they act to modulate leaf morphogenesis. In these later-formed leaves, a small decrease in the abundance of miR156/miR157 produces a disproportionately large increase in SPL activity, primarily as a result of the increased translation of SPL transcripts. Our results provide a new view of vegetative phase change in *Arabidopsis* and the mechanism by which miR156 and miR157 regulate this process.

## Introduction

Leaves produced at different times in shoot development are often morphologically distinct. In *Arabidopsis*, for example, successive rosette leaves differ in size, length:width ratio, the angle of the leaf base, hydathode number, the complexity of the vascular system, cell size, sensitivity to gibberellic acid, and the absence vs. the presence trichomes on the abaxial surface of the leaf blade [1–7]. Some of these so-called "heteroblastic" traits change gradually throughout shoot development, others change early in shoot development and are then expressed more-or-less uniformly, while still others are present at one stage of development and absent at a different stage. These latter two patterns allow the shoot to be divided into several discrete phases, the transition between which is termed "vegetative phase change" [8].

miR156 is the master regulator of vegetative phase change in *Arabidopsis* [3,9] and other flowering plants [10–15]. It is initially expressed at a very high level, and declines as the shoot develops [3,9,16–18]. This decrease is associated with an increase in the expression of its targets, *SQUAMOSA PROMOTOR BINDING PROTEIN-LIKE* (*SPL*) transcription factors, and is responsible for the transition to the adult phase. Most species also possess another miRNA, miR157, that differs from miR156 at 3 nucleotides [19]. miR157 has the same targets as miR156 and produces an over-expression phenotype similar to that of miR156 [20]. However, the normal function of miR157 is still unknown.

Although it is clear that miR156, and possibly miR157, regulate many of the changes that occur during shoot development, the function of these miRNAs at specific times in development and in specific leaves is poorly understood. In particular, it remains to be determined if miR156 is responsible for the graded changes in leaf morphology that occur during the juvenile phase and, if so, how it produces this variation. It is also important to determine if miR156 plays a role in shoot morphogenesis during the adult phase. Although miR156 is present at lower levels in the adult phase than in the juvenile phase, a comparison of the expression patterns of miR156-sensitive and miR156-insensitive SPL reporters suggests that miR156 represses *SPL* gene expression during both phases, albeit to different extents [21]. Finally, it is important to determine the mechanism by which miR156 represses gene expression. Previous studies have shown that miR156—as well as several other plant miRNAs (reviewed in [22])—mediates both transcript cleavage and translational repression [23–27], but the relative importance of these processes for the activity of miR156 remains to be determined. This question is of particular interest in light of the observation most SPL transcripts change very little during shoot development, despite the significant decrease in miR156 that occurs during this process [21].

To address these questions, we characterized the morphological and molecular phenotype of loss-of-function mutations in *MIR156* and *MIR157* genes, and measured the absolute amount of miR156/miR157 in successive leaf primordia. We also quantified the effect of varying miR156/miR157 levels on the expression of their *SPL* targets. Our results demonstrate that miR156 and miR157 have different expression patterns, different activity, and mediate transcript cleavage and translational repression to different extents at different *SPL* genes. We also show that variation in the level of miR156/miR157 only has a significant effect on *SPL* gene expression when these miRNAs are present at relatively low levels. These results provide a foundation for detailed studies of the molecular mechanism of miR156/miR157 activity and their role in shoot morphogenesis.

## Results

### *MIR156A*, *MIR156C*, *MIR157A* and *MIR157C* are the major sources of miR156 and miR157

In *Arabidopsis*, miR156 is encoded by 8 genes and miR157 is encoded by 4 genes. We characterized the contributions of these genes to the overall pool of miR156/miR157 by sequencing small RNAs from the *FRI FLC* and *FRI flc-3* genotypes [28,29]. We chose these genotypes because they represent common genotypes in naturally-occurring accessions of *Arabidopsis* [30], and because the vegetative and flowering phenotype of *FRI flc-3* is nearly identical to that of wild-type Col [2, 31]. Sequencing of small RNAs from 11-day-old shoot apices (2 replicates of each genotype) revealed an abundant 20 nt transcript that maps to *MIR156A*, *B*, *C*, *D*, *E*, and *F*, an abundant 21 nt transcript that maps to *MIR157A*, *B*, and *C*, a moderately abundant 21 nt transcript that maps to *MIR156D*, and 3 rare transcripts that map uniquely to *MIR156G*, *MIR157D* and *MIR156H* (Table 1). Unexpectedly, miR157-related transcripts were more abundant than miR56-related transcripts.

**Table 1:** miR156- and miR157-related transcripts in 11-day-old *FRI FLC* and *FRI flc-3* seedlings.

																						Number of reads per 5 million^2^	
Genes	Sequence (5' to 3')^1^																					*FRI FLC*	*FRI flc-3*
*MIR156A*,*B*,*C*,*D*,*E*,*F*		U	G	A	C	A	G	A	A	g	A	G	A	G	u	G	A	G	C	A	C	79,471	72,771
*MIR156D*	u	U	G	A	C	A	G	A	A	g	A	G	A	G	u	G	A	G	C	A	C	9,097	16,880
*MIR156G*		c	G	A	C	A	G	A	A	g	A	G	A	G	u	G	A	G	C	A	C	47	56
*MIR157A*,*B*,*C*	u	U	G	A	C	A	G	A	A	t	A	G	A	G	a	G	A	G	C	A	C	181,052	181,776
*MIR157D*	c	U	G	A	C	A	G	A	A	t	A	G	A	G	a	G	A	G	C	A	C	87	128
*MIR156H*	u	U	G	A	C	A	G	A	A	a	A	G	A	G	a	G	A	G	C	A	C	45	52

^1^Nucleotides that differ between transcripts are indicated in lower case.

^2^Average of two libraries per genotype.

To determine which genes produce these transcripts, we identified T-DNA insertions in *MIR156A*, *MIR156C*, *MIR156D*, *MIR157A*, and *MIR157C*, and used site-directed mutagenesis to produce mutations in *MIR156B*. RT-qPCR analysis of these alleles demonstrated that they eliminate or greatly reduce the primary transcripts of the affected genes (S1 Fig). We then examined the amount of miR156 and miR157 in these stocks by hybridizing RNA blots with probes for miR156, miR157, and a combination of both probes. We used this approach instead of RNA sequencing because libraries constructed with two different RNA adaptors revealed that different miRNAs ligate with different efficiencies to each adaptor [32]. Although the miR156 and miR157 probes cross-hybridize to some extent, the source of the hybridization signal could by determined by comparing the effect of *mir156* and *mir157* mutations on these signals.

**Fig 1:**
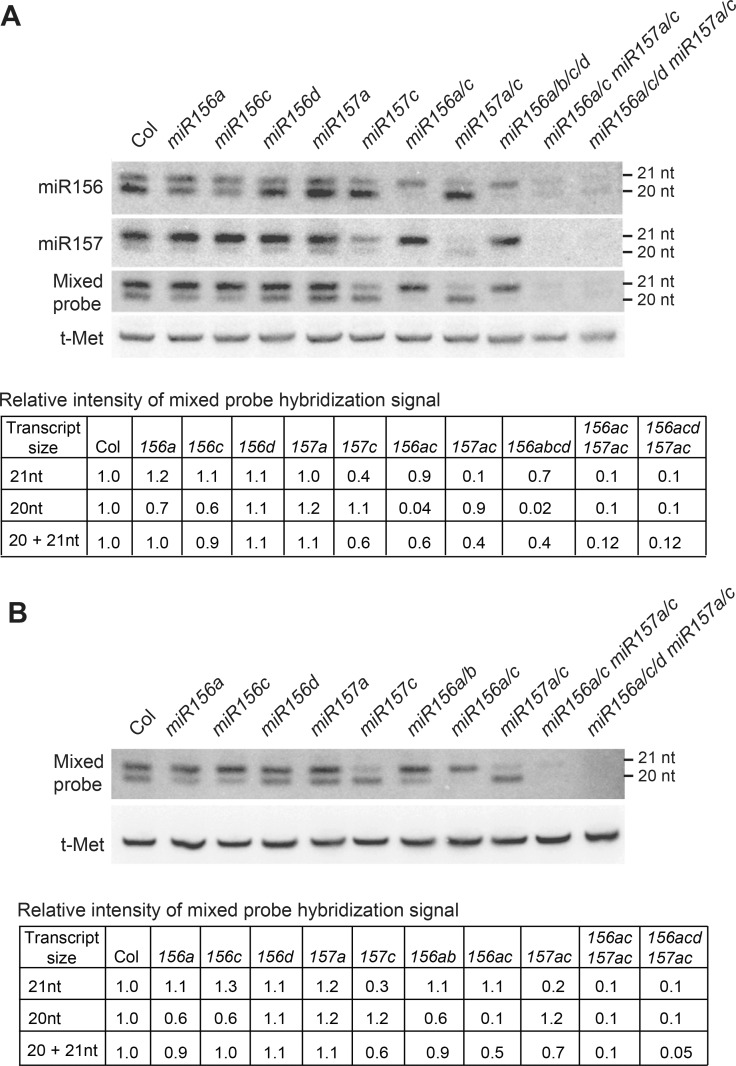
Genes contributing to the production of miR156 and miR157 in vegetative shoots. (A) Northern blot analysis of miR156 and miR157 levels in the shoot apices of 11-day-old wild-type Col and *mir156/mir157* mutants grown in LD. In Col, the miR156 and miR157 probes hybridize to 21 and 20 nt bands. A comparison of the effect of different mutations on the intensity of these bands indicates that the 20 nt transcript is miR156 and the 21 band is primarily miR157, with a small contribution from miR156d. The intensity of the 20 nt and 21 nt bands on the blot probed with a 1:1 mixture of the miR156 and miR157 probes was quantified by normalizing the intensity of each band to t-Met, and then to Col; these data are shown in the table below the figure. (B) Northern blot analysis of miR156 and miR157 levels in LP1&2 of wild-type and mutant plants grown in SD. The blots were hybridized with 1:1 mixed miR156 and miR157 probes, and the results were quantified as described above.

The effect of *mir156* and *miR157* mutations on the levels of miR156 and miR157 in 11-day-old seedlings and in 1mm primordia of leaves 1 & 2 is shown in Fig 1. In Col, the miR156 probe hybridized to 20 nt and 21 nt transcripts, with the 20 nt transcripts being more abundant than the 21 nt transcripts (Fig 1A). The abundance of the 20 nt transcripts was reduced to 62 ± 10% (± SD, n = 4) of wild-type in *mir156a-2* (hereafter, *mir156a*), to 51 ± 8% (± SD, n = 4) of wild-type in *mir156c-1* (hereafter, *mir156c*), and to 11 ± 1% (± SD, n = 3) of wild-type in the *mir156a/c* double mutant (Fig 1A). These genes are therefore the major source of the 20 nt miR156 transcripts. *mir156d-1 (*hereafter, *mir156d)* had very little effect on the overall abundance of miR156 in 11 day-old seedlings and leaf primordia (Fig 1). However, the intensity of the 21 nt band was slightly reduced in *mir156d-1* and in genotypes containing this mutation; for example, the 21 nt miR156-hybridizing band was slightly less intense in the *mir156a/c/d mir157a/c* pentuple mutant than in the *mir156a/c mir157a/c* quadruple mutant (Fig 1A). *MIR156B* also makes a minor contribution to the miR156 pool because the intensity of the miR156-hybridizing bands was essentially identical in the *mir156a/b/c/d* and *mir156a/c* mutants (Fig 1A), and there was no detectable difference between the intensity of the 20 nt and 21 nt bands in leaf primordia (LP) of the *mir156a* and *mir156a/b* mutants (Fig 1B).

In wild-type Col, the miR157 probe hybridized strongly to 21 nt transcripts and more weakly to 20 nt transcripts (Fig 1A). The 20 nt band was absent in *mir156a/c*, and thus represents cross hybridization of the miR157 probe with miR156. The intensity of the 21 nt miR157-hybridizing band was reduced to 81 ± 13% (± SD, n = 4) of wild-type in *mir157a-1*, to 26 ± 5% (± SD, n = 7) of wild-type in *mir157c-1* (*mir157c*), and to 13 ± 2% (± SD, n = 3) of wild-type in the *mir157a/c* double mutant. The remaining 21 nt signal in the *mir157a/c* double mutant partly reflects cross-hybridization of the miR157 probe with 21 nt miR156 transcripts because the intensity of this band was slightly reduced in the *mir156a/c mir157a/c* and *mir156a/c/d mir157a/c* mutants compared to *mir157a/c*. These results demonstrate that the major miR157 transcript is 21 nt, and that *MIR157C* is the major source of this transcript.

Hybridization with a 1:1 mixture of miR156/miR157 probes revealed that 21 nt transcripts are significantly more abundant than 20 nt transcripts in 11 day-old seedlings and in the primordia of leaves 1&2 (Fig 1A and 1B). The 21 nt band was reduced significantly in *mir157a/c*, and therefore corresponds primarily to miR157, whereas the 20 nt band was nearly absent in *mir156a/c*, and therefore corresponds to miR156. These results are consistent with the results of RNA sequencing (Table 1), and demonstrate that miR157 is more abundant than miR156 in young seedlings. Northern analysis using a mixed miR156/miR157 probe revealed that the amount of miR156 and miR157 in the *mir156a/c/d mir157a/c* pentuple mutant is about 10% of the wild-type level (Fig 1A and 1B). Assuming that the mutations present in this pentuple mutant are null alleles, the amount of miR156/miR157 in this line represents the combined output of *MIR156E*,*F*,*G*,*H* and *MIR157B*,*D*. These six genes therefore contribute relatively little to the production of miR156 and miR157 in seedlings.

### The expression patterns and developmental functions of miR156 and miR157

**Fig 2:**
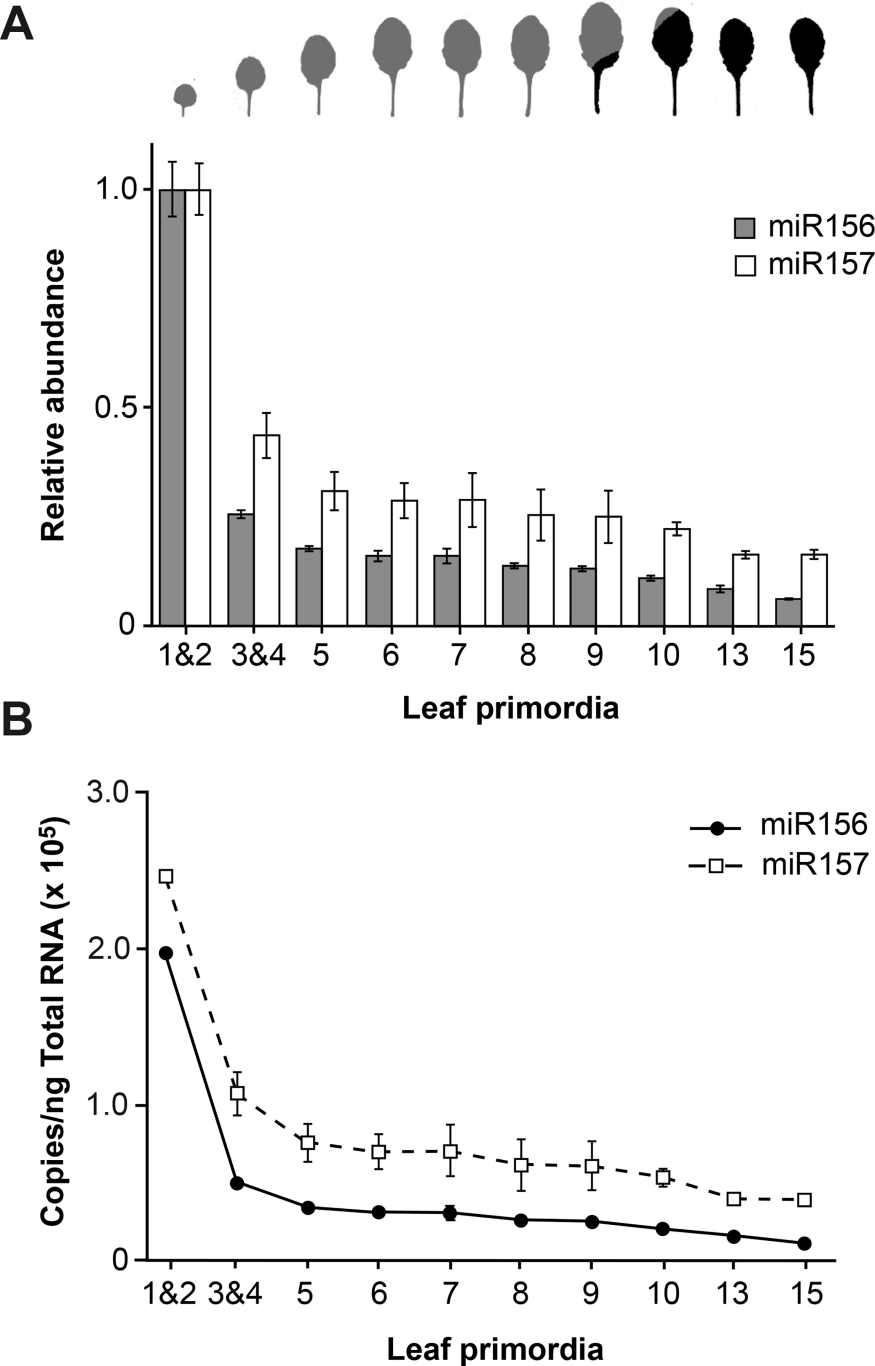
miR157 is more abundant than miR156 and declines more slowly during shoot development. (A) RT-qPCR analysis of miR156 and miR157 levels in successive leaf primordia of plants grown in SD. Values were normalized to the value in LP 1&2, and are the average of 3 biological replicates ± standard deviation. The shape of fully expanded leaves at the corresponding positions is shown at the top of the graph. Grey = no abaxial trichomes; Black = abaxial trichomes. (B) The estimated amount of miR156 and miR157 in successive leaf primordia. These values were calculated using experimental data for LP1&2, and the RT-qPCR data shown in (A).

The morphology of rosette leaves changes qualitatively and quantitatively during shoot development. In plants grown in SD to delay flowering, the first two rosette leaves are small and round, and lack serrations and abaxial trichomes [1,2] (Fig 2A). Leaves 3 and 4 are larger than leaves 1 and 2, but also have round leaf blades with no serrations and no abaxial trichomes. Leaves 5 through 9 are larger, more elongated, and more serrated than the first four leaves. Depending on light quantity and quality, abaxial trichome production begins between leaf 7 and 9, and is accompanied by a decrease in the angle of the leaf base and by the production of more prominent serrations (Fig 2B). Previous studies have shown that the juvenile forms of these traits require the activity of miR156/miR157 [9], but the relationship between the abundance of these miRNAs and the changes in leaf morphology that occur during shoot development is still unknown. To begin to answer this question, we measured the abundance of miR156 and miR157 in successive rosette leaves of wild-type plants, and characterized the effect of *mir156* and *mir157* mutations on leaf morphology.

RT-qPCR (S2A Fig) and Northern analysis (S2B Fig) demonstrate that miR156 and miR157 increase as leaves expand. However, the expression pattern of these miRNAs in successive fully expanded leaves (S2C and S2D Fig) and 1 mm LP (Fig 2) is quite similar, indicating that the factors responsible for variation in the expression of miR156/miR157 during shoot development operate at all stages of leaf development. Consistent with our previous analyses of shoot apices [21], miR156 and miR157 decrease significantly from LP1&2 to LP3&4, and then decline more gradually before reaching a relatively constant level around leaf 13 (Fig 2). LP3&4 had approximately 25%, LP9 had 12%, and LP13 had 8% of the amount of miR156 present in LP1&2. miR157 declined to a lesser extent: LP 3&4 had approximately 50%, LP9 had 25%, and LP13 had 17% of the amount of miR157 present in LP1&2 (Fig 2). The expression pattern of miR156 in fully-expanded (FE) leaves matched its expression pattern in LP, but miR157 did not decline as dramatically between FE1&2 and FE3&4 as it did between LP1&2 and LP3&4 (S2C and S2D Fig).

We then determined the absolute amount of these miRNAs in LP by comparing the RT-qPCR results obtained with leaf samples to the results obtained using known quantities of miR156 and miR157. Synthetic miR156 and miR157 transcripts were serially diluted in 600ng/μl E.coli RNA, and a standard curve was produced by plotting the concentrations of these miR156 and miR157 standards against 2^-ct^ of the corresponding RT-qPCR reaction. RT reactions were performed in parallel using 600ng of total RNA from LP1&2. The 2^-ct^ value of the LP1&2 sample was then fitted to the standard curve, and the concentration of miR156 or miR157 was calculated using linear regression. This information, and the results of the experiment shown in Fig 2A, were then used to calculate the absolute amount of miR156 and miR157 in other LP (Fig 2B). miR156 was present in LP1&2 at a concentration of 1.96 ± 0.1 x 10^5^ copies per ng total RNA, whereas miR157 was present at a concentration of 2.45 ± 0.2 x 10^5^ copies per ng total RNA (Fig 2B). miR156 subsequently declined to approximately 2.6 x 10^4^ copies per ng total RNA in LP9, whereas miR157 declined to 6.1 x 10^4^ copies per ng total RNA. Thus, the transition between leaves 1&2 and leaves 3&4 is accompanied by a major decline in the level of miR156 and miR157 whereas subsequent changes in leaf morphology are associated with much smaller changes in the abundance these miRNAs. The juvenile-to-adult transition occurred during the period when miR156 and miR157 were declining very gradually, and was accompanied by a relatively small change in the abundance of these transcripts.

**Fig 3:**
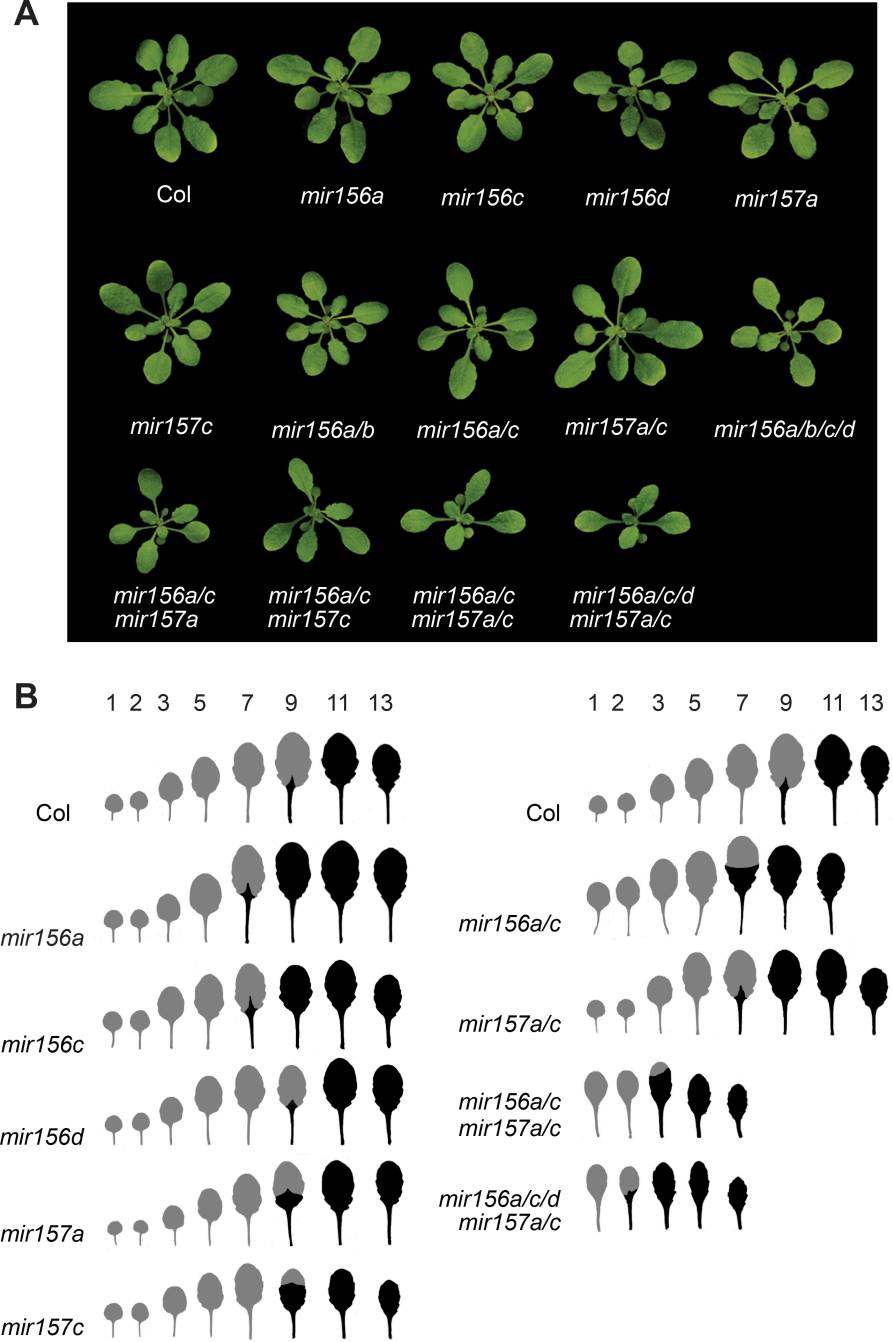
The phenotype of plants mutant for genes encoding miR156 and miR157. (A) Rosettes of three-week-old Col, and *miR156* and *miR157* single and multiple mutant plants grown in SD. (B) Representative scans of fully expanded rosette leaves 1 through 13 from Col and the *miR156* and *miR157* single and multiple mutant shown in (A). Grey = no abaxial trichomes; Black = abaxial trichomes.

**Fig 4:**
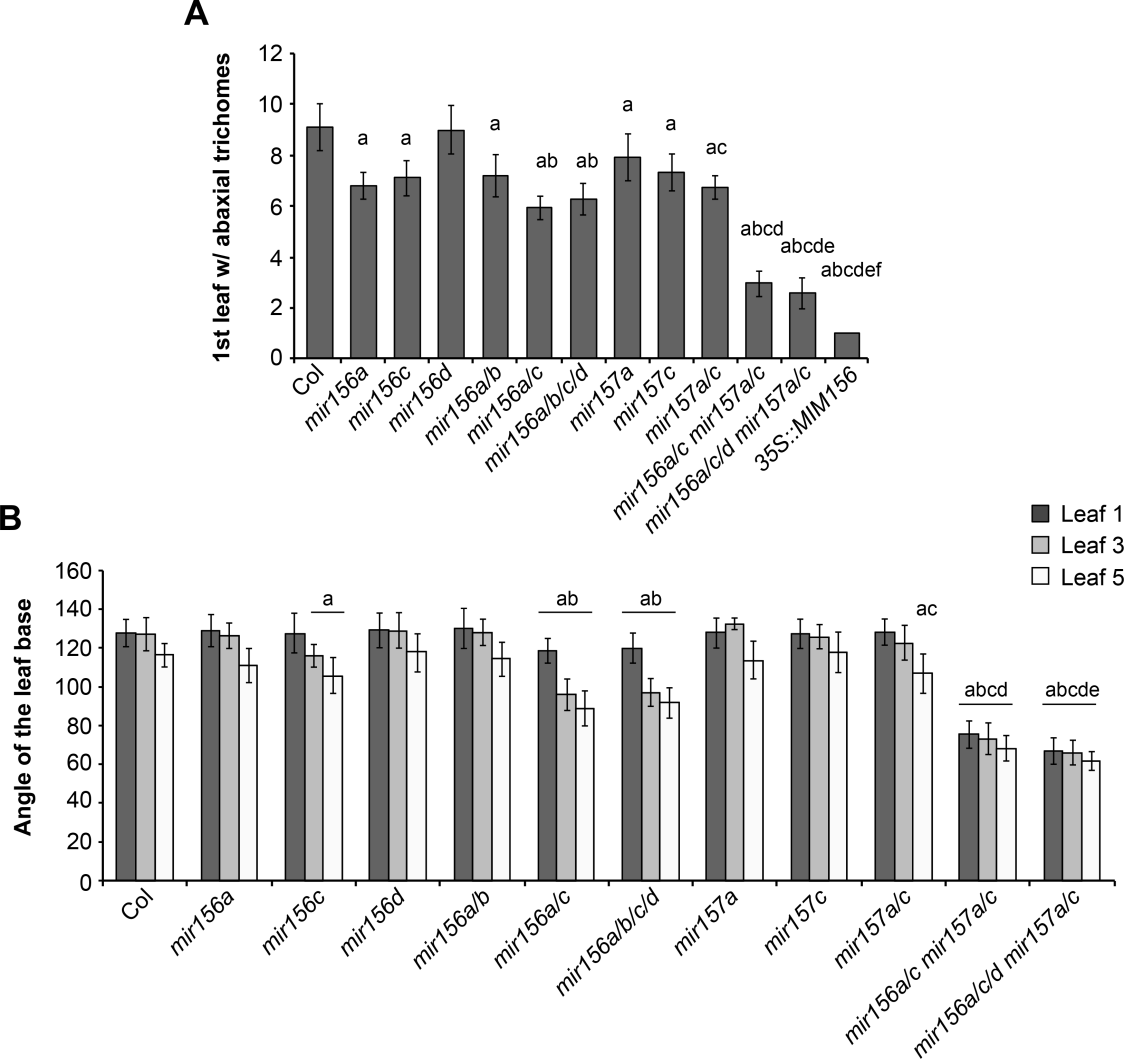
The effect of *miR156* and *miR157* mutations on leaf morphology. (A) The number of leaves without abaxial trichomes in single and multiple *mir156* and *mir157* mutants grown in SD. (B) The angle between lines tangent to leaf base in single and multiple *mir156* and *mir157* mutants grown in SD. a = significantly different from Col; b = significantly different from *mir56a* and *miR156c*; c = significantly different from *miR15a* and *miR157c;* d = significantly different from *miR156a/c* and *miR157a/c*; e = significantly different from *miR156a/c mir157a/c*; f = significantly different from *mir156a/c/d mir157a/c* (Student's t test; p < 0.01; n = 18 to 23). Error bars = standard deviation.

The relative importance of different *MIR156* and *MIR157* genes in shoot development was determined by characterizing the morphological phenotype of plants singly or multiply mutant for *mir156a*, *mir156b*, *mir156c*, *mir156d*, *mir157a* and *mir157c* (Figs 3 and 4). Plants were grown in SD to eliminate the effect of floral induction on leaf morphology [2]. We measured two traits that change with leaf position—the production of trichomes on the abaxial surface of the leaf blade and the angle of the leaf base. In wild type plants, the angle of leaf base became more acute starting with leaf 5, and abaxial trichome production started at leaf 9 (Figs 3 and 4).

The effect of *mir156* and *mir157* mutations on abaxial trichome production and leaf shape was correlated with abundance of miR156/miR157 in different leaves. Leaves produced early in shoot development, which have a relatively high level of miR156/miR157, were less sensitive to these mutations than leaves produced later in shoot development, which have a relatively low level of miR156/miR157 (Figs 3 and 4). For example, *mir156a*, *mir157a*, *mir157c*, and *mir157a/c* caused abaxial trichomes to be produced on leaves 7 and/or 8, but did not affect abaxial trichome production or the shape of leaf 1, 3, and 5. *mir156c* produced abaxial trichomes on leaves 7 and 8 and significantly reduced the angle of the leaf base in leaves 3 and 5, but had no effect on leaf 1. *miR156a/c* and *mir156a/b/c/d* reduced the angle of the leaf blade in leaves 1, 3 and 5, but had a more significant effect on leaves 3 and 5 than on leaf 1; these genotypes only produced abaxial trichomes on leaves 6 and above. *miR156a/c mir157a/c* and *mir156a/c/d mir157a/c* had a significant effect on the shape of leaves 1, 3, and 5, but rarely produced abaxial trichomes on leaf 2, and never produced abaxial trichomes on leaf 1 (Figs 3 and 4). The absence of abaxial trichomes on leaves 1 and 2 is attributable to the small amount of miR156/miR157 remaining in these mutants because *35S*::*MIM156* consistently produced abaxial trichomes on both of these leaves (Fig 4A). These results demonstrate that abaxial trichome production is more sensitive to miR156/miR157 than leaf morphology, and is strongly repressed by even low levels of these miRNAs. They also reveal that the amount of miR156/miR157 in leaves 1&2 far exceeds the amount required to specify their identity. Only genotypes with very low levels of miR156/miR157 (e.g., *miR156a/c mir157a/c*, *mir156a/c/d mir157a/c*, *35S*::*MIM156)* cause these leaves to resemble adult leaves (Figs 3B and 4B).

In general, the morphological phenotype of *mir156/mir157* mutations was correlated with their effect on the abundance of miR156 or miR157. *mir156a* and *mir156c* have a relatively large effect on the level of mir156 (Fig 1) and also have a relatively large effect on shoot morphology. *mir156c* has a more significant effect on the morphology of leaves 3 and 5 than *mir156a* (Fig 4B), which is consistent with its slightly larger effect on the abundance of miR156 (Fig 1A). *mir156b* and *mir156d* have very minor effects on the abundance of mir156 (Fig 1) and also have minor effects on shoot morphology; *mir156b* did not significantly enhance the phenotype of *mir156a* or *mir156a/c*, and *mir156d* only produced a significant effect on leaf morphology in combination with *mir156a/c* and *mir157a/c*. The only unexpected result was the phenotype of *mir157a/c*. miR157 is more abundant than miR156 and was therefore expected to play a larger role in vegetative phase change than miR156. However, *mir157a/c* had a significantly weaker effect on abaxial trichome production and leaf shape than *mir156a/c* (Figs 3 and 4), even though these double mutants have approximately the same amount of miR157 and miR156, respectively (Fig 1). This observation demonstrates that miR157 is less important for vegetative phase change than miR156, and suggests that it may be less active than miR156.

### Is miR157 less active than miR156?

**Fig 5:**
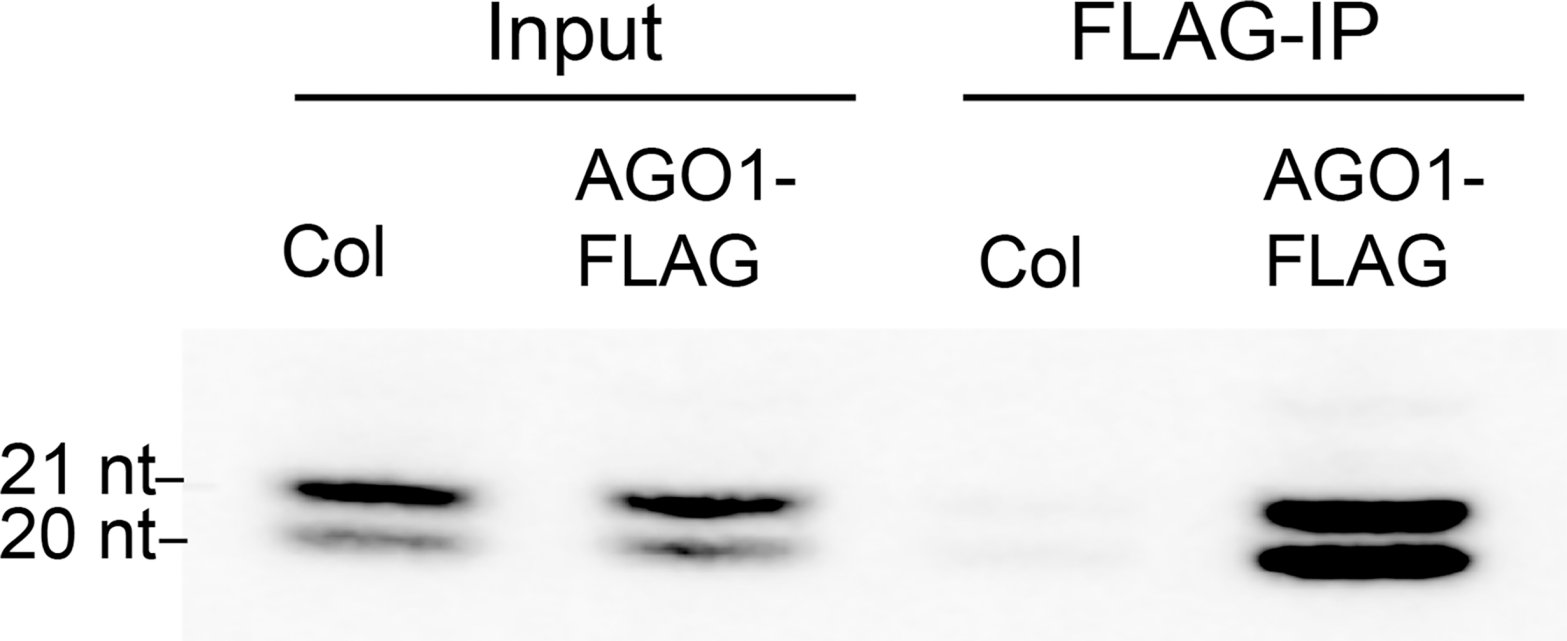
miR156 is more efficiently loaded into AGO1 than miR157. AGO1-FLAG was immunoprecipitated (IP) from *AGO1-FLAG*/*ago1-36* and Col (negative control) plants using a FLAG antibody. Small RNAs were then extracted from both the IP and non-IP fractions and subjected to Northern analysis using a mixed miR156/miR157 probe. miR157 (21 nt) was more abundant than miR156 (20 nt) in the input fraction, whereas miR156 and miR157 were about equally abundant in the IP fraction from *AGO1-FLAG*/*ago1-36* plants.

miRNAs with a 5’ terminal uridine, such as miR156 and miR157, repress the expression of their targets via their association with AGO1 [33]. To determine if miR156 and miR157 are loaded onto AGO1 with different efficiencies, we measured the amount of miR156 and miR157 associated with AGO1 *in planta*. For this purpose, we took advantage of an *ago1-36* line transformed with *AGO1-FLAG* [34]. Extracts from 2-week-old seedlings of this transgenic line and wild-type Col (as negative control) were treated with an antibody to the FLAG epitope, and small RNAs were extracted from immunoprecipitad (IP) and non-IP samples and assayed using Northern blots. Hybridization with a mixed miR156/miR157 probe revealed that miR157 (21 nt band) was more abundant than miR156 (20 nt band) in the input fraction, but that miR156 was as abundant as miR157 in the IP fraction (Fig 5). This result indicates that miR156 is more efficiently loaded onto AGO1 than miR157.

**Fig 6:**
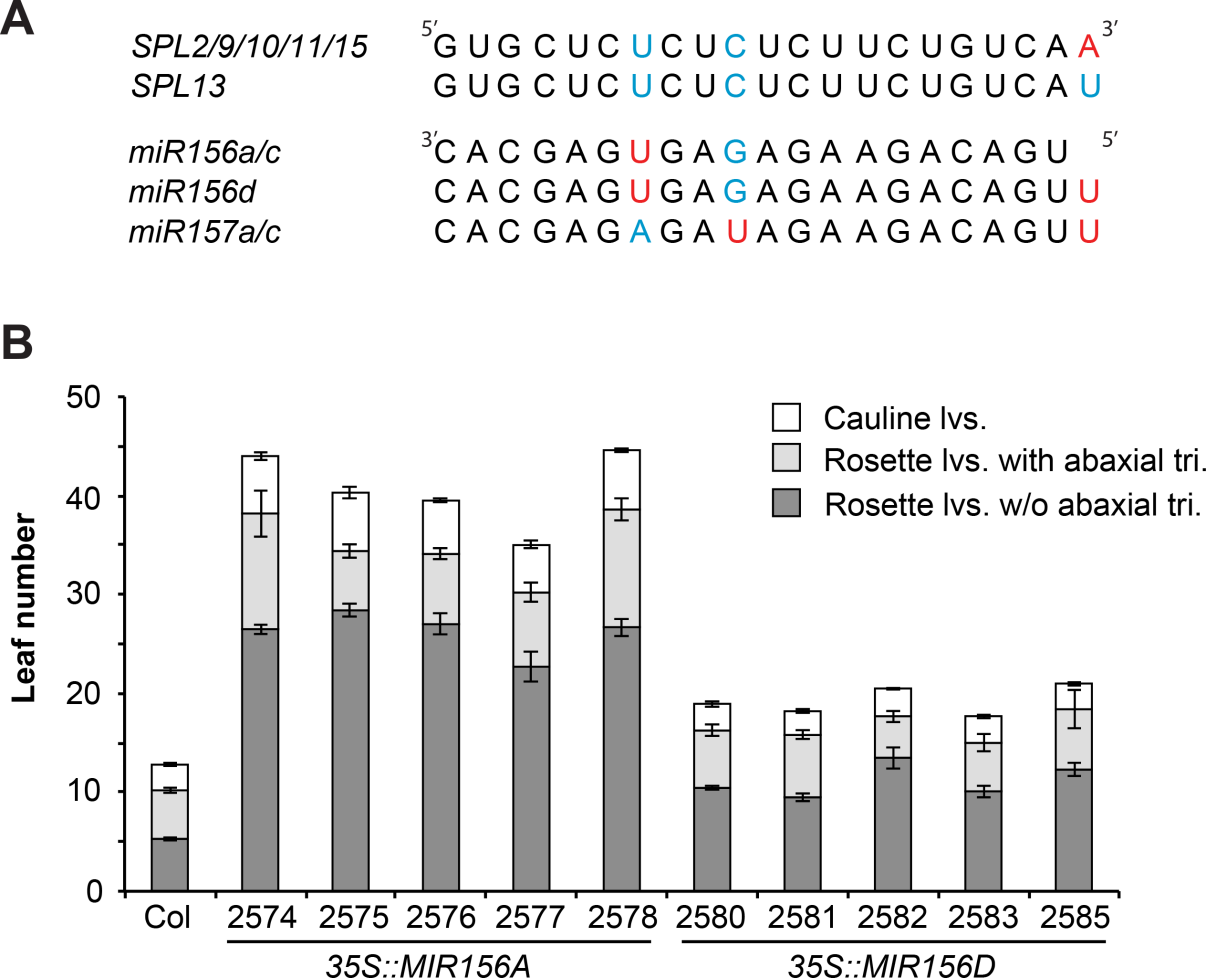
miR156d is less effective than miR156a. (A) The sequence of miR156 and miR157 transcripts and their target site in SPL transcripts. Only the SPL transcripts involved in vegetative phase change [21] are shown. Nucleotides that are mis-paired in the miR156/miR157:SPL duplex are shown in different colors. (B) The phenotype of transgenic lines expressing *MIR156A* or *MIR156D* under the regulation of the CaMV 35S promoter. Lines over-expressing miR156a have significantly more rosette leaves without abaxial trichomes, more total rosette leaves, and more cauline leaves than lines over-expressing miR156d. n = 8 to 12, ± SD.

This cannot be the only reason for the difference in the phenotypes of *mir156a/c* and *mir157a/c* because the amount of miR156 and miR157 associated with AGO1 is quite similar. Another possibility is that the AGO1-miR157 complex is inherently less active than the AGO1-miR156 complex. miR156 and miR157 bind to the SPL2, SPL9, SPL10, SPL11, and SPL15 transcripts with only one mismatched nucleotide, although the position of this nucleotide is different for the two miRNAs (Fig 6A). In addition to two internal nucleotides, miR157 differs from miR156 in possessing an additional U at its 5' end. This 5’ U is unpaired in the miR157-SPL13 duplex (Table 1). To determine if this extra nucleotide might influence the activity of miR157 we compared the relative strengths of miR156a and miR156d. The miR156d transcript is identical to the miR156a transcript, except for the presence of an additional 5’U (Fig 6A). The phenotypes of 5 transgenic lines constitutively expressing a genomic fragment containing *MIR156A* under the regulation of the CaMV 35S promoter, and an equal number of lines containing a similar construct encoding *MIR156D* [3], were compared under LD conditions. The lines used for this analysis were selected because they possessed a single T-DNA insertion site. The *35S*::*MIR156A* lines produced approximately twice as many leaves without abaxial trichomes and approximately twice as many cauline leaves as the lines transformed with *35S*::*MIR156D* (Fig 6B). This result demonstrates that *MIR156D* is less effective than *MIR156A*, and suggests that the additional 5' U in miR157 is partly responsible for its lower biological activity.

### *SPL* transcripts are differentially responsive to miR156/miR157

**Fig 7:**
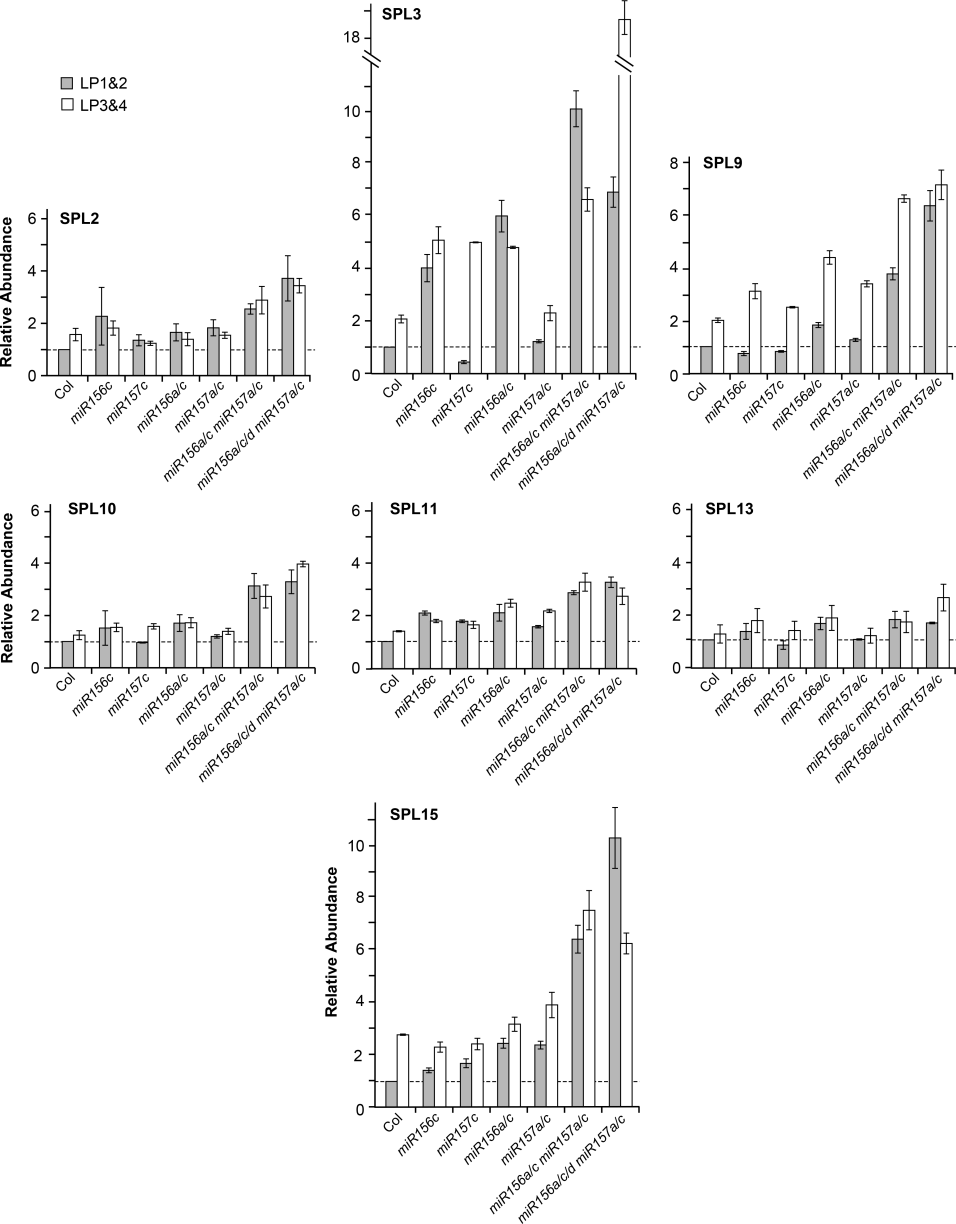
RT-qPCR analysis of SPL transcript levels in 1 mm leaf primordia of Col and *mir156/mir157* mutants grown in SD. Most transcripts increase less than 2-fold between LP1&2 and LP3&4 in Col, and increase 2-fold or less in *miR156* and *miR157* mutants. Results are relative to the value for Col LP1&2. Error bars represent the SD for 3 biological replicates.

To determine the molecular basis for the effect of *mir156* and *mir157* mutations on leaf morphology, we compared SPL transcript levels in LP1&2 and LP3&4 in wild-type and *mir156/mir157* mutant plants (Fig 7). Consistent with their modest effect on leaf morphology, single *mir156c* and *mir157c* mutations had a very small effect on SPL transcripts. However, plants with multiple *mir156* and/or *mir157* mutations displayed a significant increase in the level of some SPL transcripts. SPL3 transcripts were particularly responsive to a decrease in the level of miR156, increasing about 4-fold in *mir156c* and 5-to-6-fold in *mir156a/c*. In contrast, SPL3 transcripts were relatively insensitive to a decrease in miR157, except in genotypes that were also deficient for miR156. For example, SPL3 was elevated nearly 20 fold in LP3&4 of the *mir156a/c/d mir157a/c* pentuple mutant. SPL9 and SPL15 transcripts increased very slightly in *mir156a/c* and *mir157a/c* but increased up to 6-fold in *mir156a/c mir157a/c* and *mir156a/c/d mir157a/c*. SPL2, SPL10 and SPL11 increased 2-fold or less in *mir156a/c* and *mir157a/c*, and only about 3-fold in *mir156a/c mir157a/c* and *mir156a/c/d mir157a/c*. SPL13 transcripts were unaffected in *mir157a/c*, were elevated about 2-fold in both *mir156a/c* and *mi156a/c mir157a/c*, and were only slightly more abundant than this in *mir156a/c/d mir157a/c*.

The abundance of SPL3 is regulated directly by miR156/miR157 via miRNA-induced transcript cleavage, and indirectly by the effect of miR156/miR157-regulated SPL proteins on the expression of miR172, which in turn represses a group of *AP2*-like genes that repress the transcription of *SPL3*, *SPL4*, and *SPL5* [3,9,23,35]. This combination of direct post-transcriptional regulation by miR156/miR157 and indirect transcriptional regulation via the miR172-AP2 pathway may be responsible for the hypersensitivity of SPL3 transcripts to variation in the abundance of miR156/miR157. In contrast, the only way in which miR156/miR157 have been found to regulate the expression of other *SPL* genes is through a direct interaction with their transcripts. These findings therefore suggest that the SPL2, SPL9, SPL10, SPL11, SPL13 and SPL15 transcripts are differentially sensitive to destabilization by miR156 and miR157.

### miR156/miR157 are responsible for the temporal expression pattern of most *SPL* genes

**Fig 8:**
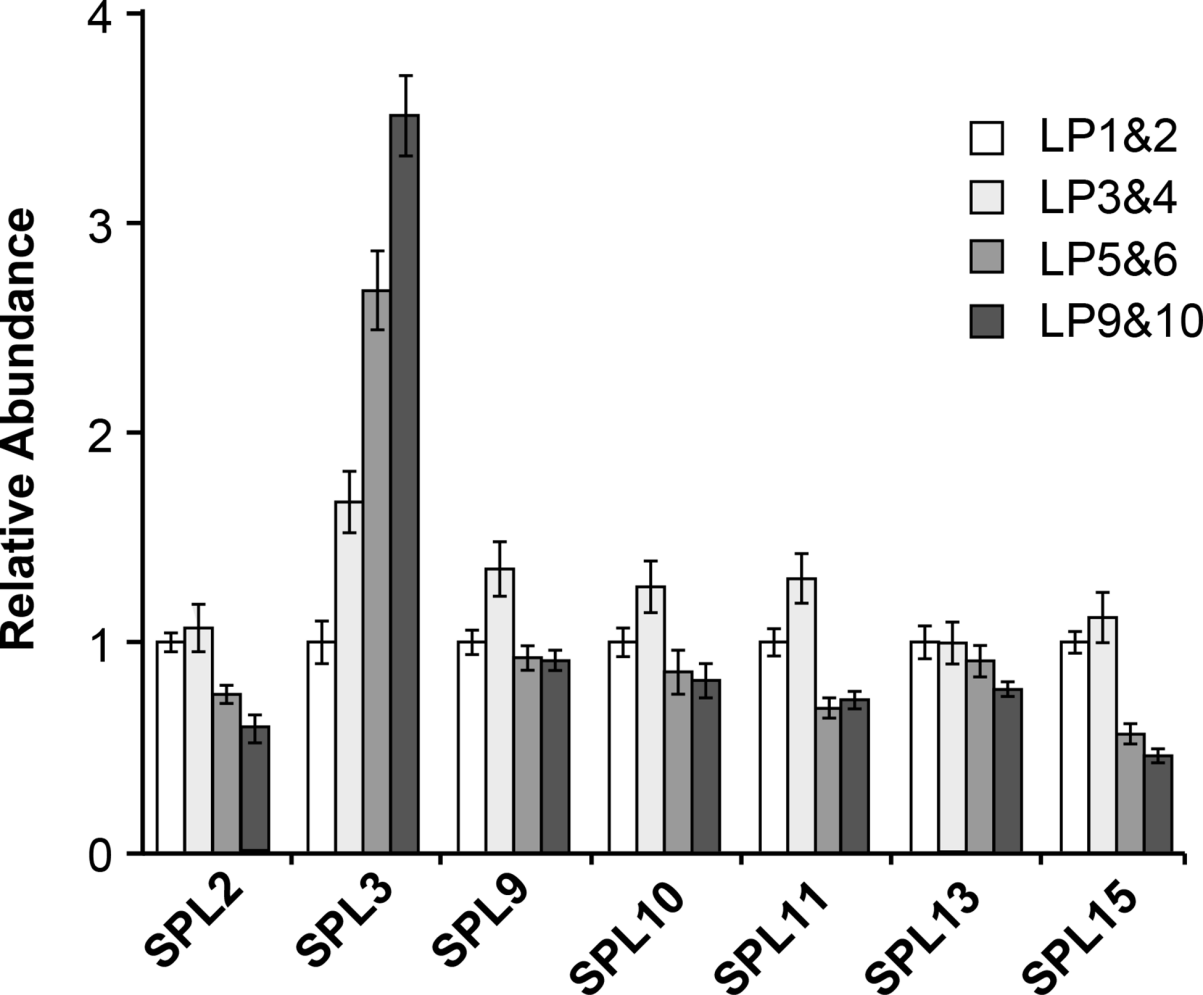
RT-qPCR analysis of SPL transcript levels in successive 1 mm leaf primordia of the *mir156a/c mir157a/c* mutant. Error bars represent the SD for 3 biological replicates.

Both the transcripts and the protein products of miR156/miR157-regulated *SPL* genes increase during shoot development [3,18,21,36,37]. The expression patterns of miR156/miR157-resistant reporter genes suggest that this increase is largely mediated by miR156/miR157 [21], but whether miR156/miR157 are entirely responsible for the temporal expression pattern of *SPL* genes is still unknown. To answer this question, we measured the abundance of the SPL2, SPL3, SPL9, SPL10, SPL11, SPL13 and SPL15 transcripts in successive leaf primordia of the *mir156a/c mir157a/c* mutant (Fig 8). Most of these transcripts were present at either the same level or at slightly lower levels in adult LP (LP5,6,9,10) compared to juvenile LP (LP1,2,3,4). The only exception was SPL3, which increased 3–4 fold from LP1&2 to LP9&10. This result suggests that miR156/miR157 are entirely responsible for the temporal increase in the SPL2, SPL9. SPL10, SPL11, SPL13 and SPL15 transcripts, whereas the temporal increase in SPL3 transcripts may be partly regulated by factors that operate independently of miR156/miR157. Alternatively, the temporal increase in SPL3 may be attributable to the small amount of miR156/miR157 remaining in this quadruple mutant.

### *SPL9* and *SPL13* are essential for the expression of adult vegetative traits

**Fig 9:**
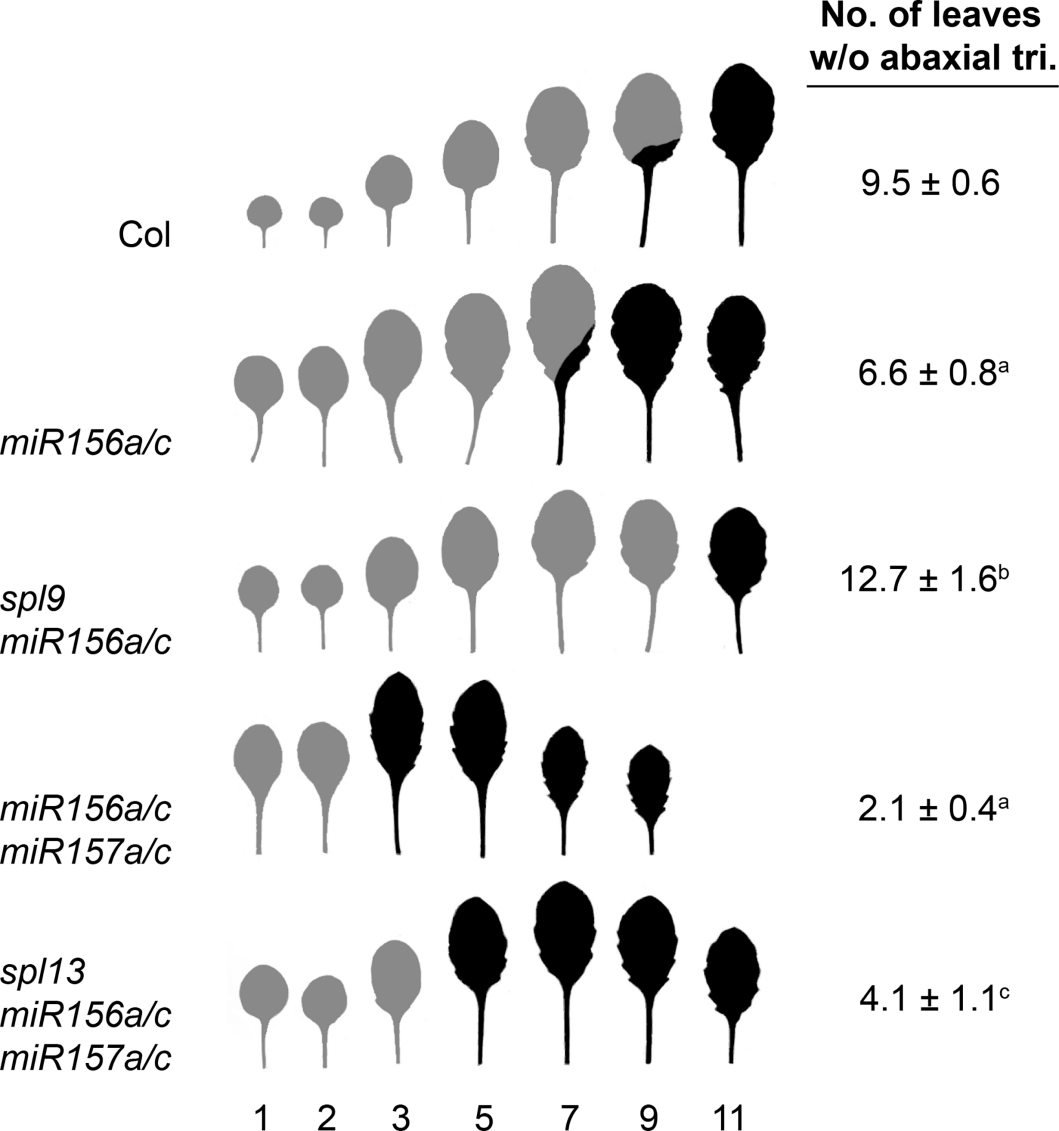
*SPL9* and *SPL13* contribute to the precocious phenotype of *mir156* and *mir157* mutants. The morphology of fully expanded leaves and the number of leaves without abaxial trichomes in Col and mutant plants grown in SD. Grey = no abaxial trichomes; Black = abaxial trichomes. a = significantly different from Col; b = significantly different from *mir156a/c*, c = significantly different from *miR156a/c mir157a/c*. Student's T-test, ± SD.

The degree to which the abundance of different SPL transcripts changes in response to changes in the level of miR156/miR157 does not necessarily reflect the developmental importance of these *SPL* genes because miR156/miR157 can mediate both transcript cleavage [3,38–40] and translational repression [23–26]. For example, *SPL3* is highly expressed in vegetative shoots and is more sensitive to miR156/miR157 than any other *SPL* gene, but the phenotype of *spl3* mutations demonstrate that it plays little or no role in vegetative development [21]. We were particularly interested in determining whether *SPL9* and *SPL13* contribute to the precocious phenotype of *mir156/mir157* mutants because SPL9 transcripts increase as miR156/miR157 levels decline, whereas SPL13 transcripts are relatively insensitive to changes in these miRNAs (Fig 7). To address this question, we introduced *spl9* into a *mir156a/c* mutant background and introduced *spl13* into a *mir156a/c mir157a/c* mutant background. *spl9* completely suppressed the precocious abaxial trichome phenotype and partially suppressed the leaf shape phenotype of *mir156a/c*, whereas *spl13* partially suppressed the effect of *mir156a/c mir157a/c* on both of these traits (Fig 9). Thus, *SPL9* and *SPL13* both play important roles in miR156-mediated developmental transitions.

### miR156/miR157 regulate *SPL9* and *SPL13* by different mechanisms

**Fig 10:**
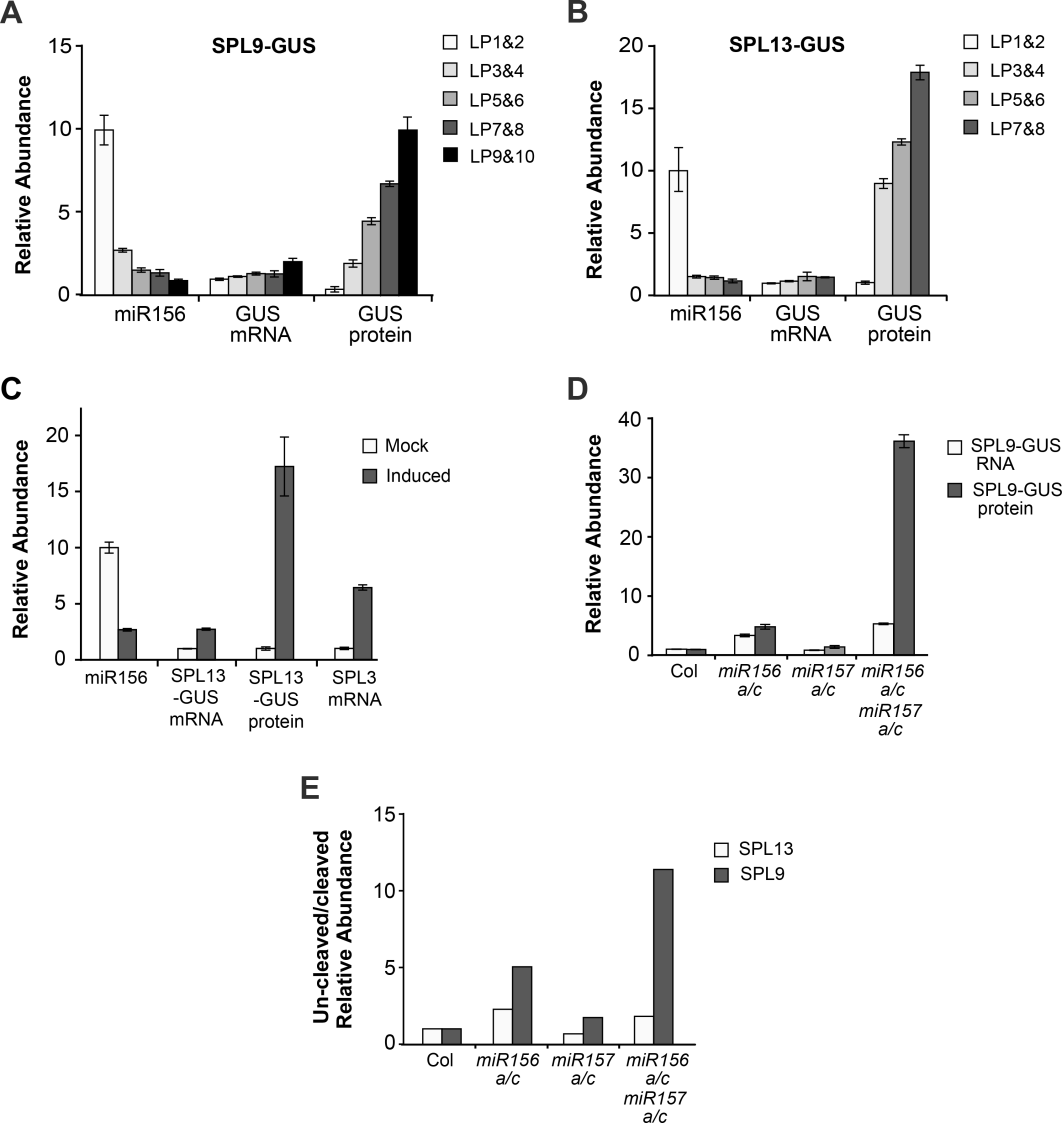
Developmental variation in SPL9 and SPL13 protein levels is mediated primarily by miR156/miR157-directed translational repression. (A) Developmental variation in miR156, SPL9-GUS mRNA, and SPL9-GUS protein in leaf primordia of Col plants grown in SD. miR156 and SPL9-GUS transcripts were measured by RT-qPCR, while the abundance of the SPL9-GUS protein was determined using the MUG assay for GUS activity. Values were normalized to the level in LP1&2, which was set to 1 for SPL9-GUS, and 10 for miR156. Data are the average of 3 biological replicates, ± SD. (B) Developmental variation in miR156, SPL13-GUS mRNA, and SPL13-GUS protein in the leaf primordia of Col plants grown in SD; data analysis as in (A). (C) Quantitative analysis of the effect of an induced reduction in miR156 on the level of SPL13-GUS mRNA and protein. The *SPL13-GUS* line was crossed to the *Ind-MIM156 line*, and these transgenes were then made homozygous. Young leaf primordia from mock-treated and β-estradiol-treated plants were harvested and analyzed by qRT-PCR and the MUG assay, as described in the Materials and Methods. SPL3 transcript levels were measured as a positive control for miR156 knock-down. (D) Quantitative analysis of SPL9-GUS mRNA (RT-qPCR) and protein (MUG assay) in Col, *miR156a/c*, *miR157a/c* and *mir156a/c mir157a/c*. (E) The relative abundance of uncleaved/cleaved SPL9 and SPL13 transcripts in LP1&2 of Col, *miR156a/c*, *miR157a/c* and *mir156a/c mir157a/c*. Values are relative to the value in Col. Results are from a single experiment with 3 technical replicates. In A, B, C and D the error bars represent the SD of 3 biological replicates.

We studied the mechanism by which miR156/miR157 regulate the expression of *SPL9* and *SPL13* by comparing the abundance of the SPL9 and SPL13 mRNAs with the abundance of their protein products. Antibodies against SPL9 and SPL13 are not available, so we used previously described [21] and newly generated *SPL9-GUS* and *SPL13-GUS* translational reporters to visualize these proteins in transgenic plants. Leaf primordia were harvested sequentially as the shoot developed, and the abundance of the SPL9-GUS, SPL13-GUS and miR156 transcripts was measured by RT-qPCR, while the abundance of the SPL9-GUS and SPL13-GUS proteins was measured using the MUG assay. Consistent with previous results [21], a nearly10-fold decrease in the level of miR156 between LP1&2 and LP9&10 was accompanied by very modest (2-fold or less) increase in the level of the SPL9-GUS and SPL13-GUS transcripts (Fig 10A and 10B). In contrast, the activity of the SPL9-GUS protein increased 10-fold between LP1&2 and LP9&10 (Fig 10A), whereas the activity of SPL13-GUS protein increased 15-fold between LP1&2 and LP7&8 (Fig 10B). The relationship between the change in miR156 levels and the change in SPL9-GUS and SPL13-GUS expression varied from leaf to leaf. The 4-fold decrease in miR156 between LP1&2 and LP3&4 was associated with a 3-fold increase in SPL9-GUS activity and a 9-fold increase in SPL13-GUS activity, but subsequent smaller changes in miR156 were associated with disproportionately large increases in the expression of these reporters. For example, in the SPL9-GUS line, miR156 declined by about 2-fold between P3&4 and LP9&10, while the amount of SPL9-GUS protein increased 9-fold. In the SPL13-GUS line, miR156 declined by only 10% between LP3&4 and LP7&8, while the amount of SPL13-GUS protein doubled.

To examine the quantitative relationship between miR156 and *SPL13* expression in more detail, we took advantage of a transgenic line containing an estrogen-inducible miR156 target-site mimic (*Ind-MIM156*), which enabled us to decrease the activity of miR156 by exogenous application of β-estradiol. One-week-old plants homozygous for the *SPL13-GUS* and *In-MIM156* transgenes were given mock and β-estradiol treatments, and LP1&2 were harvested 24 hours later and analyzed by RT-qPCR and the MUG assay. This treatment reduced the abundance of miR156 by about 3-fold and produced a 2-fold increase in SPL13-GUS mRNA, but increased the abundance of the SPL13-GUS protein by greater than 15-fold (Fig 10C). Because the amount of active miR156 in *In-MIM156* may not be measured accurately by RT-qPCR, we also examined the abundance of SPL3 transcripts in mock- and estradiol-treated plants. The abundance of SPL3 mRNA is hypersensitive to variation in miR156 and thus serves as a proxy for the abundance of miR156 (Fig 7). SPL3 transcripts were 4-fold more abundant in induced plants relative to mock-treated plants (Fig 10C), which is similar to difference in the amount of SPL3 transcripts in Col vs. *mir156c* (Fig 7). *mir156c* reduces miR156 by about 50% (Fig 1B). Consequently, this result implies that estradiol-treated plants had approximately 50% less active miR156 than mock-treated plants, which is consistent with amount of miR156 detected by RT-qPCR. In summary, these results provide further evidence that miR156/miR157 regulate the expression of SPL13 primarily by promoting its translational repression, and also demonstrate that SPL13 activity responds non-linearly to changes in the abundance of these miRNAs.

SPL9 transcripts are more sensitive to changes in miR156/miR157 than SPL13 transcripts (Fig 7), suggesting that transcript cleavage may play a larger role in the regulation of *SPL9* than *SPL13*. To address this possibility, we introduced the miR156-sensitive *SPL9-GUS* reporter into *mir156a/c*, *mir157a/c*, and *mir156/c mir157a/c* mutant backgrounds, and measured the abundance of the SPL9-GUS mRNA and protein in LP1&2. *mir157a/c* did not have a significant effect on SPL9-GUS mRNA or protein levels, but *mir156a/c* produced a 2-fold increase in the SPL9-GUS transcript and a 5-fold increase in the SPL9-GUS protein (Fig 10D). *mir156a/c mir157a/c* had an even more dramatic effect on the expression of *SPL9-GUS*, producing a 4-fold increase in the SPL9-GUS transcript and an ~36 fold increase in the SPL9-GUS protein (Fig 10D). These results demonstrate that miR156/miR157 repress *SPL9* both by destabilizing the SPL9 transcript and by repressing its translation. The increase in *SPL9* activity that occurs during shoot development [21] is probably attributable primarily to a reduction in miR156/miR157-mediated translational repression because the SPL9-GUS protein increases more significantly in response to a decrease in miR156/miR157 than the SPL9-GUS transcript.

To compare the sensitivity of the SPL9 and SPL13 transcripts to miR156/miR157-mediated cleavage, we used a modified form of 5’ RNA Ligase Mediated Rapid Amplification of cDNA Ends (5’ RLM-RACE) [3,41] to quantify the ratio of un-cleaved/cleaved SPL9 and SPL13 transcripts in wild-type Col and mutants deficient for miR156 and miR157. Equal amounts of total RNA from LP1&2 were ligated to a 5’-end RNA adaptor, and the purified RNA ligation products were then used in RT reactions using a poly-T primer. The levels of un-cleaved and cleaved SPL transcripts were then measured by qPCR, using primers specific for each type of transcript. These results were normalized to elf4A1, and the un-cleaved/cleaved transcript ratio in each genotype was then calculated by dividing the relative expression values. This ratio does not necessarily reflect the actual difference between these transcripts because primers for un-cleaved and cleaved transcripts may have different amplification efficiencies. Consequently, instead of using this ratio to compare the relative abundance of cleaved SPL9 and SPL13 transcripts, we asked whether the cleavage of these transcripts is differentially sensitive to variation in the level of miR156/miR157. This was done by normalizing the un-cleaved/cleaved transcript ratio from different mutants to the value in Col. The ratio of un-cleaved:cleaved SPL13 transcripts was about 2-fold greater in *mir156a/c* and *mir156a/c mir157a/c* than in Col, whereas the ratio of un-cleaved:cleaved SPL9 transcripts was 5-fold greater in *mir156a/c* and 15-fold greater in *mir156a/c mir157a/c* than in Col (Fig 10E). Thus, SPL9 is more sensitive than SPL13 to miR156/miR157-directed transcript cleavage.

### How much miR156 is required to repress *SPL* expression?

**Fig 11:**
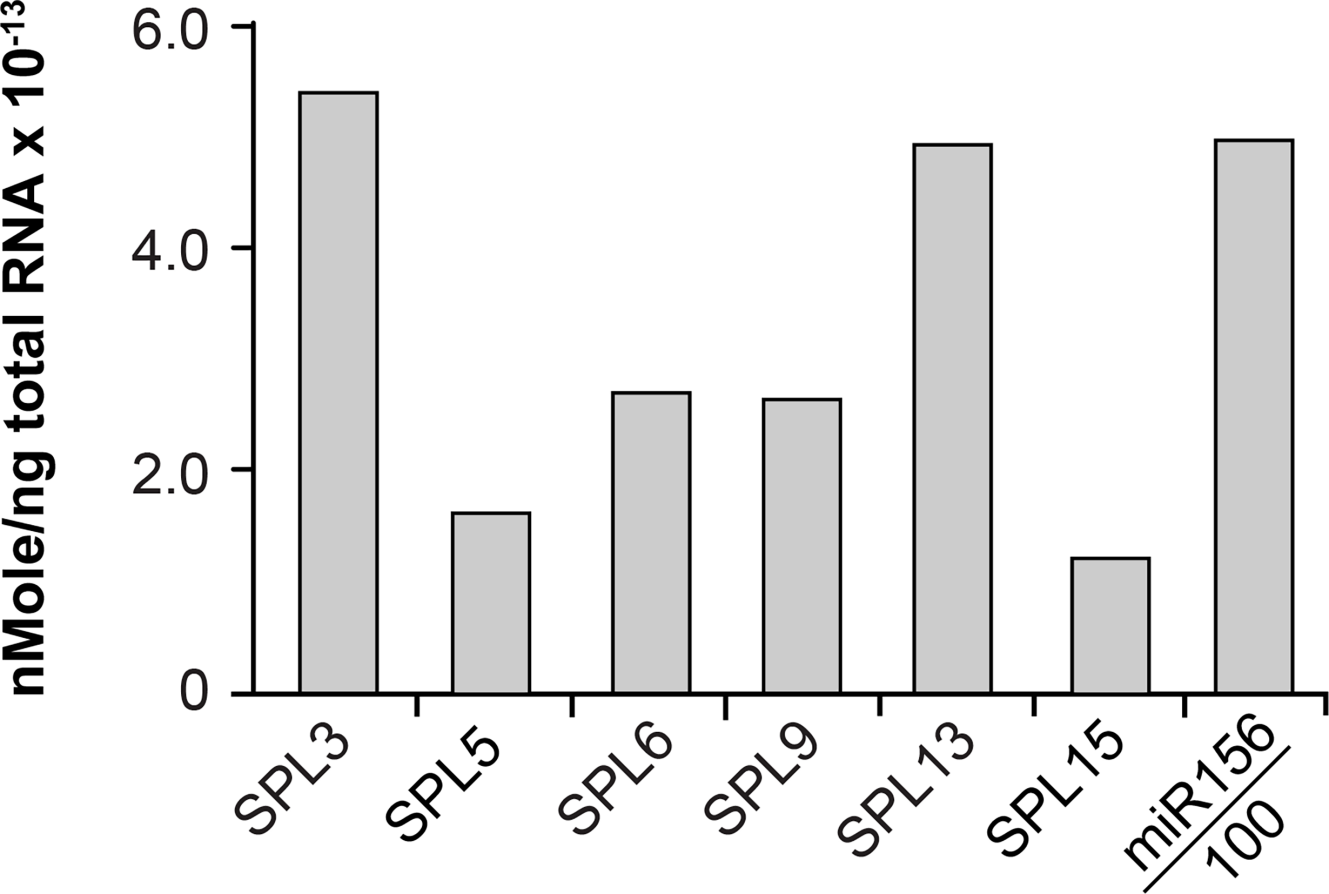
miR156 is 100 times more abundant than its SPL targets. The absolute abundance of miR156 and SPL transcripts in Col LP3&4. The value shown for miR156 is 0.01 of the actual value.

The stoichiometry of a miRNA and its target can influence the mechanism of gene silencing [42]. To determine if the mode of action of miR156 is related to the relative abundance of miR156 and its targets, we measured the absolute quantity of several SPL transcripts and miR156 in LP3&4—the leaves in which the translational reporters for *SPL3*, *SPL9*, and *SPL13* are first expressed [21]. This was done using known concentrations of SPL transcripts and miR156 as standards, and performing RT-qPCR on these standards in parallel with RNA from LP3&4. There was a 5-fold range in the abundance of different SPL transcripts, with SPL5 and SPL15 being the least abundant, and SPL3 and SPL13 being the most abundant (Fig 11). miR156 was 100 times more abundant than SPL3 and SPL13, about 200 times more abundant than SPL6 and SPL9, and about 500 times more abundant than SPL5 and SPL15 (Fig 11). This result therefore suggests that greater than a 100-to-200-fold excess of miR156 is required to completely repress *SPL* genes. Assuming that the transcription rate of these *SPL* genes is the same in LP1&2 and LP3&4, we predict that the amount of miR156 in LP1&2 (where all miR156-regulated genes are completely repressed [21]) is approximately 300–600 times greater than the amount of SPL3 and SPL13 transcripts, and approximately 1,500 times greater than the amount of SPL5 and SPL15 transcripts. Although the relative abundance of miR156 vs. SPL9 and SPL13 might suggest that translational repression is favored by a relatively low miR156:SPL transcript ratio (SPL13) whereas transcriptional cleavage is favored by a high miR156:SPL transcript ratio (SPL9), this seems unlikely because a 90% reduction in the level of miR156 in *mir156a/c* produced only a slight increase in the level most SPL transcripts, including SPL9 (Fig 6). Indeed, we only observed a major increase in SPL transcripts in the *mir156a/c mir157a/c* quadruple mutant, implying that transcript cleavage does not require high levels of these miRNAs. Thus, the miR156/SPL transcript ratio cannot explain the difference in the sensitivity of the SPL9 and SPL13 transcripts to miR156-directed translational repression.

## Discussion

The phenotype of plants over-expressing miR156 or miR157 reveals that these miRNAs promote the juvenile vegetative phase, but how they specify heteroblastic patterns of leaf morphology, as well as the individual functions *MIR156* and *MIR157* genes, are unknown. We addressed these questions by accurately measuring the abundance of miR156 and miR157 in leaves with different developmental identities, and by characterizing the phenotype of plants deficient for different *MIR156* and *MIR157* genes. We found that miR156 and miR157 have similar—but not identical—expression patterns, and that miR157 is more abundant, but less effective, than miR156. We also found that heteroblastic variation in leaf morphology is correlated with the relative abundance of miR156/miR157, and that different features of leaf morphology are differentially sensitive to the level of these miRNAs. Our observation that variation in the abundance of miR156/miR157 produces non-linear changes in the protein level of their targets suggests a molecular mechanism for the qualitative and quantitative changes in leaf morphology that occur during shoot development.

### The nature of vegetative phase change

The vegetative period of shoot development is typically divided into two phases—a juvenile phase and an adult phase. However, many species display considerable morphological variation during these phases. In some species the first few leaves are referred to as "seedling leaves" because they are anatomically or morphologically distinct from other juvenile leaves [43–47]. In *Arabidopsis*, leaves 1&2 differ from other juvenile leaves in that they are smaller and rounder, have a less complex vascular system, and are less sensitive to exogenously applied gibberellin than other juvenile leaves [1,2,6]. Leaves 1&2 also have much lower levels of SPL proteins than other juvenile leaves [21]. We found that leaves 1&2 have a significantly more miR156/miR157 than other juvenile leaves, and that the largest absolute as well as relative decrease in these miRNAs occurs between leaves 1&2 and leaves 3&4. Additionally, the amount of miR156/miR157 in leaves 1&2 far exceeds the amount that is actually required to determine their identity; mutations that nearly completely eliminate miR156 have a similar effect on leaves 3 and 5, but a much weaker effect on leaf 1. The only genotypes that caused leaf 1 to resemble leaves 3 and 5 were those that reduce miR156/miR157 by 90%. These results suggest that leaves 1&2 represent a distinct developmental phase.

Starting with leaf 3, leaf size, the number of leaf serrations, and the angle of the leaf base change gradually from leaf-to-leaf. These gradual changes in leaf morphology are accompanied by an increased ability to produce abaxial trichomes [1], which first appear between leaf 6 to 9. In contrast to the morphological stability of leaves 1&2, the morphology of these “late” juvenile leaves is influenced by light intensity, photoperiod, and the reproductive state of the shoot [2, 5]. This combination of quantitative and qualitative changes, as well as the morphological plasticity of late juvenile leaves, can be explained by the relatively low and gradually decreasing level of miR156/miR157 in successive leaves, and by the non-linear response of some *SPL* genes to changes in the abundance these miRNAs. Features of leaf morphology that change continuously from leaf-to-leaf are likely to be controlled by pathways or processes whose activity is directly correlated with the level of *SPL* gene expression, whereas all-or-none traits, such trichome initiation, may only appear when the expression of these genes exceeds a threshold.

Our results also explain why the expression of phase-specific traits becomes dissociated under certain conditions. For example, abaxial trichome production is more responsive to conditions that promote floral induction than either hydathode number or leaf shape [2]. Juvenile and adult vegetative traits can also be dissociated in English ivy, resulting in plants that display different combinations of these traits [48,49]. We suspect that this phenomenon is attributable to functional differentiation between *SPL* genes, coupled with variation in their sensitivity to miR156 and miR157. Some *SPL* genes are expressed at relatively high levels and respond nearly linearly to changes in the level of miR156/miR157, whereas others are expressed at relatively low levels and only respond significantly to a change in the level of miR156/miR157 when these miRNAs are present at very low levels. Given that *SPL* genes are not functionally identical [9,21,50], conditions that produce small changes in miR156/miR157, or which elevate the transcription of particular *SPL* genes above the threshold established by miR156/miR157, could lead to unusual combinations of phase-specific traits.

### The developmental and molecular functions of miR156 and miR157

miR156 is one of the oldest and most highly conserved miRNAs in plants [51,52]. Although miR157 is nearly as old and as highly conserved as miR156, this is not widely appreciated because miR157 is frequently annotated as miR156 in small RNA sequencing studies and in miRBase (http://www.mirbase.org). The failure to distinguish these miRNAs is likely based on the assumption that they have the same function. Our comparison of the expression patterns of miR156 and miR157, as well the phenotype of plants lacking one or both of these miRNAs, demonstrates that these miRNAs work together to regulate vegetative phase change, but are not functionally identical. miR157 is more abundant than miR156 and is expressed in a similar temporal pattern, but miR156 plays a more important role in vegetative phase change and is a more potent repressor of *SPL* gene expression. The difference in the activity of these miRNAs may be due, in part, to the lower efficiency with which miR157 is loaded onto AGO1. However, this is not the only reason for the difference in their activity because the amount of miR157 associated with AGO1 is not dramatically lower than the amount of miR156 associated with AGO1. Another factor that may contribute to the difference in their activity is the structure of the miRNA:target-site duplex [41,53–55]. miR156 and miR157 bind to most of their targets with a single mismatch, but this mismatch is located one nucleotide from the cleavage site in the case of miR157 and 3 nucleotides from the cleavage site in the case of miR156. miR157 also has an additional 5' nucleotide (relative to miR156), which is unpaired in the miR157:SPL13 duplex. *SPL13* plays a major role in vegetative phase change [21] and if this mismatch reduces the ability of miR157 to repress the activity of *SPL13*, this would be expected to have a significant phenotypic effect. We suspect that this extra 5' uracil is primarily responsible for the relatively low activity of miR157 because miR156d also has an extra 5' uracil and is significantly less active than miR156, despite being otherwise identical to miR156. However, we cannot rule out the possibility that the difference in the activity of miR156 and miR157 is a consequence of the difference in their length, rather than the specific features of the miRNA:SPL duplex. Most miRNAs in plants are 21 or 22 nt in length, but several evolutionarily conserved miRNAs are 20 nt, or exist as both 20 nt and 21 nt variants [52,56]. miRNAs that are 22 nt are uniquely capable of generating tasiRNAs and other types of phased siRNAs [57,58], but it is still unknown if 20 and 21 nt miRNAs are functionally distinct. The 20 nt miR156 transcript is present in the moss, *Physcomitrella patens* [59,60], and in virtually all other plants that have been examined to date [51,52]. miR157 is absent in *Physcomitrella*, but is present in *Selaginella* and most, but not all, higher plants [61]. The fact that miR157 has been conserved along with miR156 during plant evolution suggests that it is not completely redundant with miR156, and raises the possibility that the relative activity of these miRNAs may differ in different species.

### The source of miR156 and miR157

The effect of mutations in different *MIR156* and *MIR157* genes on the abundance of miR156 and miR157 demonstrates that *MIR156A* and *MIR156C* produce most of the miR156 in the shoot whereas *MIR157C* produces most of the miR157. *MIR156D* and *MIR157A* are expressed at a much lower level than these loci, but the ability of *mir156d* and *mir157a* to enhance the phenotype of plants mutant for *mir156a*, *mir156c*, and *mir157c* demonstrates that they are functionally significant. We do not know if the miR156 and miR157 transcripts that remain in the *mir156a/c/d mir156a/c* mutant are derived from one or more these loci or from other *MIR156/MIR157* genes because we cannot be certain that the mutations present in this mutant stock are completely null. Whatever the case, these remaining transcripts are functionally active because the phenotype of this pentuple mutant is not as strong as the phenotype of plants over-expressing a miR156 target site mimic [62]. A complete picture of the function of this gene family will require identifying loss-of-function mutations in *MIR156E*, *F*, *G* and *H*, and *MIR157B* and *D*.

### The relative importance of transcript cleavage and translational repression for the activity of miR156/miR157

The phenotype of slicer-defective AGO1 mutants suggests than plant miRNAs destabilize transcripts exclusively by transcript cleavage, and that this is the primary mode by which they regulate gene expression [63,64]. However, the results of this and previous studies [23–26] indicate that miR156 and several other plant miRNAs act primarily by promoting translational repression [24–27,65,66]. This observation begs the question of why translational repression is so important for the function of these miRNAs, and how the choice between transcript cleavage and translational repression is regulated. Our results suggest that the amount of miR156 and miR157 present in both juvenile and adult leaves is sufficient to almost completely saturate the cleavage machinery at most of their targets. However, the response of different SPL transcripts to miR156/miR157 varies between transcripts, suggesting that the susceptibility of these transcripts to miR156/miR157-induced cleavage depends on sequences outside the miR156/miR157 target site. In plants, the importance of sequences flanking a miRNA target site has been demonstrated for miR159 [42,67] and for several miRNA-cleaved transcripts that generate phasiRNAs [68]. However, it is unclear if the sequence environment of a miRNA target site influences the mechanism by which a miRNA represses gene expression. A comparison of the molecular mechanism by which miR156/miR157 regulate the expression of *SPL9* and *SPL13* will be informative because these genes respond very differently to changes in the level of these miRNAs. miR156/miR157 are among the oldest miRNAs in plants, and it is therefore reasonable to conclude that miRNA-induced translational repression is an ancient regulatory mechanism in plants. Identifying the biochemical factors that induce AGO1 to direct transcript cleavage vs. translational repression, and defining the functional consequences of these modes of regulation, are important problems for future research.

## Materials and methods

### Plant materials and growth conditions

All of the lines used in this study were in a Col genetic background. The *mir156a-2* and *mir156c-1* mutations have been described previously [69]. *mir156d-1* (SALK_40772), *mir157a-1* (Flag_375C03), *mir157c-1* (SALK_039809) were obtained from the *Arabidopsis* Biological Resource Center (Ohio State University, Columbus, OH) and were crossed to Col at least 3 times before further analysis. *mir156b-1* was generated by TALEN-directed mutagenesis [70] in a *mir156c-1* background, and is a 42 nt-deletion within the MIR156B hairpin sequence (AACAGAGAAAACTGACAGAA—-42 bp deletion—GCGTGTGCGTGCTCACCTCTC) that removes most of the miR156 sequence. Multiple mutant lines were generated by inter-crossing mutations and then screening F2 populations for the desired genotypes using the allele-specific primers listed in S1 Table. Seeds were sown on Farfard #2 Mix and placed at 4° C for 3 days before moving to a Conviron growth chamber, where they were grown under either long day (16 hrs light/8 hrs dark; 80 μmol m^-2^ s^-1^) or short day (10 hrs light/ 14 hrs dark; 130 μmol m^-2^ s^-1^) conditions, with illumination provided by a 6:2 ratio of broad spectrum (Interlectric Tru-lite) and red light-enriched (Interelectric Gro-lite) fluorescent lights.

### Transgenic plants

The miR156-sensitive and miR156-resistant *SPL13-GUS* reporter lines used in this study were described previously [21]. The previously described *SPL9-GUS* reporter lines [21] silenced when they were crossed into a *miR156a-2* background, so it was necessary to produce new lines for these reporters. For this purpose, miR156-sensitive and miR156-resistant *SPL9*:*SPL9-GUS* genomic sequences [21] were inserted into the pCAM-NAP:eGPF vector [71] using the restriction enzymes XmaI and SbfI. These constructs were then introduced into the *miR156a-2/miR156c-1 miR157a-1/miR157c-1* lines by *Agrobacterium*-mediated transformation. Homozygous single insertion lines were selected as described previously [71], and crossed to Col and further genotyped to obtain *SPL9-GUS* reporters in different genetic backgrounds.

### Estradiol-induced gene expression

The estradiol-inducible *MIM156* line (*Ind-MIM156*) was constructed using a Gateway compatible version of the XVE system, as described by Brand and colleagues [72]. The *MIM156* sequence described by Franco-Zorilla and colleagues [53] was cloned into pMDC160 by standard Gateway cloning using the primers in S1 Table (referred to as pMDC160-MIM156). Plants containing pMDC150-35S [72] were crossed to transgenic pMDC160-MIM156 plants and made homozygous. Induction of gene expression was performed by spraying 10μM 17-ß-estradiol (0.01% Silwet 77) on seedlings at the desired time point. Tissues were harvested at 24hr after induction.

### MUG assay

Tissue samples were harvested into 2ml tubes submerged in liquid nitrogen, and then homogenized using a bead-beater. 300μl of extraction buffer (10 mM EDTA pH 8.0, 0.1% SDS, 50 mM sodium phosphate pH 7.0, 0.1% Triton X-100; 10 mM ß-mercaptoethanol and 25 μg/ml PMSF added fresh before experiment) was then added to each tube. Samples were mixed well and incubated on ice for 10 mins, after which they were centrifuged at 4°C (13000 rpm) for 15 mins. to remove cell debris. 96ul of supernatant was removed and incubated with 4ul of 25mM 4-MUG at 37°C. Incubation time varied among reporters to ensure the end fluorescence readings fell within a linear range. The reaction was terminated by adding 100ul of 1M sodium carbonate to each tube, and fluorescence was measured using a Modulus fluorometer (E6072 filter kit). The amount of MU in each sample was then calculated by comparing this reading to a standard curve constructed by plotting the fluorescence readings from serial dilutions (100nM, 250nM, 500nM, 1000n) of 4-MU. The 4-MU equivalent was divided by the incubation time and this value was then normalized to the amount of protein in the sample, which was determined by performing a Bradford assay on the supernatant remaining in the original tube. For each sample, GUS activity was expressed as 4-MU equivalent/min/mg protein. Values were then normalized to the control sample of each experiment.

### RT-qPCR analysis

RNA was extracted from leaf primordia no larger than 1mm in length using Trizol (Invitrogen), and samples were then treated with DNase (Ambion) following the manufacturer’s instructions. To measure the abundance of miRNAs, 600ng of RNA was used in a reverse transcription reaction with a SnoR101 reverse primer and a miRNA-specific RT primer. To measure the abundance of SPL transcripts, 600ng RNA was used in a reverse transcription reaction primed with Oligo(dT). qPCR was performed on the resulting products, using the primers listed in S1 Table. Reactions were performed in triplicate for each biological replicate.

### Northern blotting of miRNAs

Tissue was homogenized in liquid nitrogen, and total RNA was then extracted using Trizol (Invitrogen). Extracts were incubated in 500mM NaCl and 5% PEG8000 on ice for 2 hours, and centrifuged at 13,000 rpm for 10min. The supernatant was incubated with a 10% volume of 3M NaOAc and 2 volumes of 100% ethanol at -20°C for 2 hours. Small RNAs were precipitated by centrifugation at 13,000 rpm for 10min, and washed in cold 75% ethanol twice. RNA blotting was performed as described previously [3]. A 1:1 ratio of miR156 and miR157 probes was used for mixed probe hybridizations.

### RNA sequencing

Sequencing libraries were generated from small RNAs isolated from shoot apices of *FRI FLC* and *FRI flc-3* seedlings grown in the conditions described by Willmann and colleagues [2]. The shoot apex samples consisted of the shoot apical meristem and leaf primordia 1 mm or less in length. Libraries were generated using a lab-assembled version of Illumina's 2007 small RNA library sample preparation protocol, followed by high-throughput sequencing with Illumina's Genome Analyzer II platform.

### Absolute quantification of SPL transcripts and miRNAs

The miR156 and miR157 transcripts used as references were synthesized by IDT, and the SPL transcripts used as references were synthesized by *in vitro* transcription. The template for each *in vitro* transcription reaction was generated by PCR, using the primers listed in S1 Table and cDNA from Col. Each purified SPL transcript was assayed by denaturing gel electrophoresis to confirm that the *in vitro* transcription product was a single species of the expected size. To quantify SPL transcripts, the reference mRNA generated by *in vitro* transcription was diluted to 1.00E^-8^ M and this sample was then used to create a 10x dilution series in 600ng/μl total RNA from *E*. *coli*. This dilution series was analyzed by RT-qPCR in parallel with RNA isolated from LP3&4. A series of 2x dilutions of the reference mRNA sample whose concentration was similar to that of the experimental sample was then constructed, and run along with the experimental sample in a second RT-qPCR reaction. The 2^-CT^ values of the reference samples were plotted against their known concentrations, and the CT value of the unknown sample was then placed on this graph to determine the RNA concentration.

### Modified 5’–RACE quantification of cleaved SPL transcripts

Ligation reactions were performed with 5 μg of total plant RNA and 1 μg of the GeneRacer (Invitrogen) RNA adapter following the manufacturer’s instructions, but without carrying out the de-capping reaction. After 2 hrs of incubation at 37°C, the reaction mixture was diluted with nuclease- free water and RNA was extracted in phenol: chloroform. The purified ligation product was dissolved in 10μl nuclease-free water, and 5μl of this solution was used in a reverse transcription reaction with an oligo(dT) primer. qPCR was performed using primers listed in S1 Table to quantify cleaved and un-cleaved SPL transcripts.

### Immunoprecipitation of AGO1-FLAG

Two-week-old seedlings were harvested in liquid nitrogen and homogenized in a cold motar and pestle. For each sample, approximately 1mL ground powder was dissolved in 2mL lysis buffer (50mM Tris HCl, pH 7.4, with 150mM NaCl, 1mM EDTA, 1% Triton X-100, 1mM PMSF, 1% Protease Inhibitor) followed by 15 min incubation on ice. 20% of the homogenized sample was saved for RNA extraction, and the rest was centrifuged at 13,000 rpm at 4°C for 20min to remove cell debris. The resulting supernatant was then filtered through a 45μm filter. Immunoprecipitation was performed using Anti-FLAG M2 Magnetic Beads (Sigma) following the manufacturer’s instructions. RNA was extracted from the beads using Trizol (Invitrogen) and analyzed by Northern blotting.

### Accession numbers

Small RNA sequence data are available in the NCBI Gene Expression Omnibus database under series accession number GSE72303.

## Supporting information

S1 Fig RT-qPCR analysis of the effect of *mir156* and *mir157* mutations on the abundance of the primary transcripts of the corresponding genes. (TIF)

S2 Fig The expression patterns of miR156 and miR157 during leaf and shoot development. (A) RT-qPCR analysis of the abundance of miR156 at different stages in the development of leaves 1&2. (B) Northern blot of small RNAs isolated from leaves 1&2 of 11-day-old and 21-day-old Col, *miR156a/c*, and *mir157a/c* mutants, hybridized with a mixed miR156 & miR157 probe. (C) Northern blot of small RNAs isolated from fully expanded leaves of Col, *miR156a/c*, and *mir157a/c* mutants, hybridized with a mixed miR156 & miR157 probe. Leaves were harvested immediately after they had reached full expansion. (D) Quantitation of the results shown in (C). Blots were scanned using ImageQuant, and band intensities were then normalized to t-Met, and then to the value for the 21 nt. band in Col. One lane of reach blot was loaded with a sample from a different blot to ensure that the hybridization intensity of different blots was comparable.
(TIF)

S1 Table PCR primers used in this study. (XLSX)
