# Supplementary figures and images for: Threshold-dependent repression of SPL gene expression by miR156/miR157 controls vegetative phase change in Arabidopsis thaliana
Source: PLoS Genet. 2018 Apr 19;14(4):e1007337. doi: 10.1371/journal.pgen.1007337 (PMC5929574; doi:10.1371/journal.pgen.1007337)

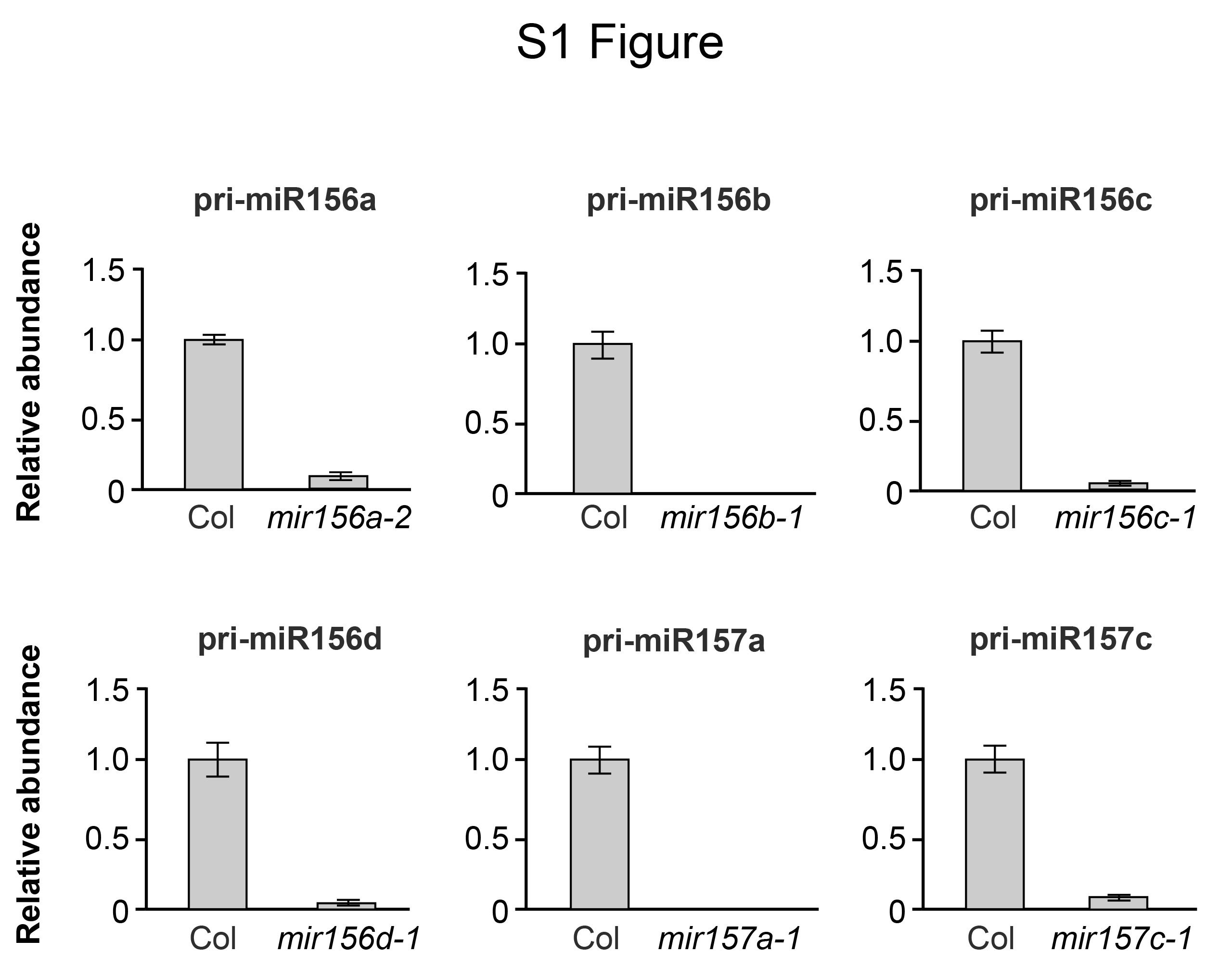

Supplement: S1 Fig — (TIF) [file pgen.1007337.s001.tif]

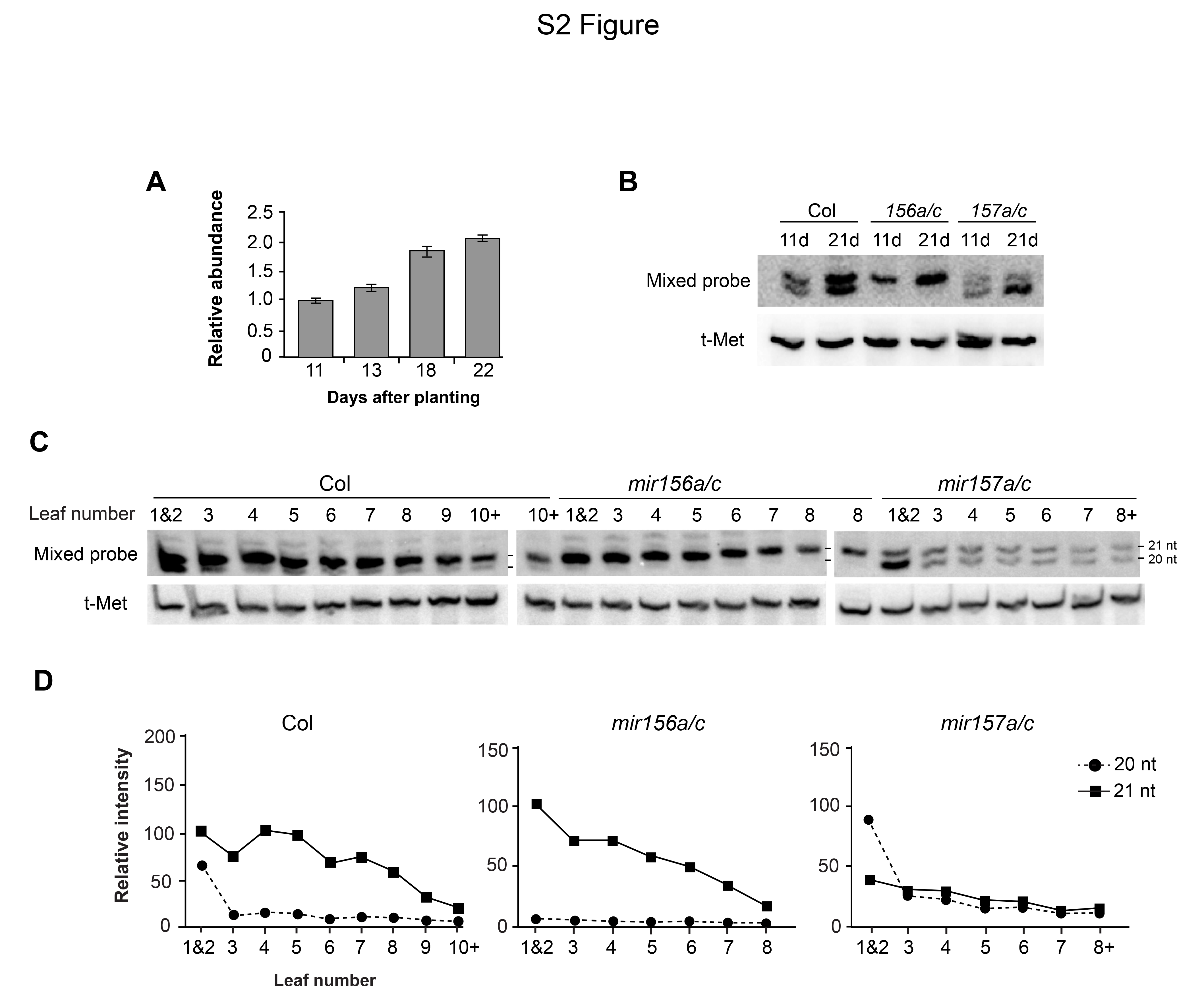

Supplement: S2 Fig — (A) RT-qPCR analysis of the abundance of miR156 at different stages in the development of leaves 1&2. (B) Northern blot of small RNAs isolated from leaves 1&2 of 11-day-old and 21-day-old Col, miR156a/c, and mir157a/c mutants, hybridized with a mixed miR156 & miR157 probe. (C) Northern blot of small RNAs isolated from fully expanded leaves of Col, miR156a/c, and mir157a/c mutants, hybridized with a mixed miR156 & miR157 probe. Leaves were harvested immediately after they had reached full expansion. (D) Quantitation of the results shown in (C). Blots were scanned using ImageQuant, and band intensities were then normalized to t-Met, and then to the value for the 21 nt. band in Col. One lane of reach blot was loaded with a sample from a different blot to ensure that the hybridization intensity of different blots was comparable. (TIF) [file pgen.1007337.s002.tif]
